# Multiple timescales of context influence perceptual sensitivity to common pairings of musical pitch and timbre

**DOI:** 10.1371/journal.pone.0328490

**Published:** 2025-07-18

**Authors:** Christian E. Stilp, Isabel Adames, Anya E. Shorey

**Affiliations:** Department of Psychological and Brain Sciences, University of Louisville, Louisville, Kentucky, United States of America; Indiana University, UNITED STATES OF AMERICA

## Abstract

Previous studies have established that musical pitch and timbre (specifically, spectral shape) perceptually covary: lower pitches are associated with darker timbres (less higher-frequency energy) and higher pitches are associated with brighter timbres (more higher-frequency energy). In four experiments, perceptual sensitivity to this relationship was assessed in pitch labeling tasks when instrument timbre varied in ways that respected or violated this pattern (Consistent or Reversed trials). Performance was influenced by context at multiple timescales: block-level (stimulus type), experimental session-level (block order or configuration), and longer-term experience (musical training background). Across experiments, participants performed near ceiling accuracy for Consistent stimuli, but were less accurate for Reversed stimuli. This pattern was moderated by which condition was tested first in the experiment, the introduction of trial-by-trial feedback, and presentation of trials in blocked versus interleaved orders. Higher musical training scores were generally associated with higher accuracy on Consistent trials but were more reliably and more strongly associated with higher accuracy on Reversed trials. Thus, context on multiple timescales can shape perceptual sensitivity to the natural covariance between musical pitch and timbre. Results advance the efficient coding hypothesis by demonstrating how listener factors can modulate perceptual sensitivity to statistical structure in the acoustic environment.

## Introduction

Natural sounds are acoustically complex, varying along many different acoustic dimensions. While myriad studies have demonstrated perceptual difficulties in the face of acoustic variability, here we focus on the fact that any such dimensions (and their respective variabilities) are not necessarily independent of one another. Studies in both speech and music perception have documented instances of perceptual interference, in which perception of a sound property or identity is impeded by concurrent variability in another feature. In speech perception, recognition of a word is impeded by variability in who spoke it [1–4] or how it was produced [5–6]. Likewise, recognition of a talker is impeded by variability in the words being spoken [1]. In music perception, recognition of the pitch of a tone or chord is impeded by variability in the instrument that produced it [7–12, but see 13–14]. Likewise again, timbre-based judgments are challenged by concurrent pitch variability [8–9, 15–16; also see 17].

According to the efficient coding hypothesis, sensory systems have adapted and evolved to be highly sensitive to structure in the sensory environment [18–19]. To mitigate the perceptual difficulties incurred by acoustic variability in a sound feature(s), here we focus on patterns of covariance among sound features. Listeners implicitly and automatically learn patterns of covariation (in some cases quite rapidly [20]), and perception often benefits when sound features respect these patterns [20–22]. Among the many examples available, here we focus on perceptual benefits from patterns of covariance involving fundamental frequency (f0) or pitch, as it is a pervasive and significant feature in speech and music alike.

Two patterns of covariation involving f0 are reviewed here. First, in speech, considerable covariance exists between f0 and formant frequencies (resonances of their vocal tract, which shift to lower or higher frequencies for longer or shorter vocal tract lengths, respectively). Across talkers, those with lower f0s tend to have longer vocal tracts and lower formant frequencies (e.g., adult cisgender men), and those with progressively higher f0s also tend to have progressively shorter vocal tracts with correspondingly higher formant frequencies (e.g., moving from adult cisgender men to adult cisgender women to children). Across all of the vowels in the Hillenbrand et al. [23] database, a vowel’s f0 and its first three formant frequencies shared an average of 77% of their variability [24]. Assmann and Nearey [25] revealed that vowels spoken by different talkers were recognized more accurately when f0 and formant frequencies respected these patterns of covariance (e.g., low f0 with lower formant frequencies) compared to when vowels violated these patterns (e.g., low f0 with higher formant frequencies).

Second, in music, a variety of studies reported that pitch perception was supported by coherent variation in instrument timbre. In discrimination tasks, pitch discrimination as well as timbre discrimination markedly improved when the other dimension was varying congruently (e.g., higher pitches occurring with brighter timbres) versus incongruently (e.g., higher pitches occurring with darker timbres [26–27]). In pitch interval estimation tasks, melodic intervals were perceived as being larger when accompanied by congruent changes in timbre (an ascending interval with the timbre moving from darker to brighter across tones) than incongruent changes (the same ascending interval with timbre moving from brighter to darker [28–29]; see also [30–31]. Additionally, under certain conditions, musical tones with coherent pairings of pitch and brightness are rated as being more pleasant than tones with incoherent pairings [32]. The association between higher pitch heights and higher vertical positions in space (Spatial-Musical Association of Response Codes, or SMARC [33–34]) only holds when pitch and brightness are covarying [35]. However, associations between pitch and timbre might vary across instruments, given differing degrees of pitch-invariance observed in studies by McAdams and colleagues [36] and by Siedenburg and colleagues [37].

Perceptual sensitivity to patterns of covariance among sound features in speech and music is likely shaped by long-term perceptual experience and/or expertise, but studying this particular influence is dotted with challenges. While one might study children’s perception to examine effects of experience, it is difficult to distinguish poorer sensitivity to stimulus covariance from immature perceptual faculties. In adulthood, perceptual faculties are functionally if not fully mature, but listeners’ incomparable experience hearing their native language(s) far exceeds conventional definitions of perceptual expertise, thus making it difficult to study effects of experience/expertise on perceptual sensitivity to the statistical regularities therein. On the other hand, musical expertise varies widely in adult listeners, making it well-suited for this research question.

To effectively examine how musical background is related to perceptual sensitivity to pitch-timbre covariance, two shortcomings require addressing. First, pitch-timbre covariance has rarely been the focus of previous investigations, which instead examined pitch perception or pitch-timbre interference. Other studies focusing on this relationship have been primarily analytic rather than primarily perceptual [37]. But, indirect evidence is available to suggest that musical training shapes perceptual sensitivity to the natural covariance between pitch and timbre. Musical training provides a degree of resilience to pitch-timbre interference [7, 9, 15; but see 26]. Other studies have either suggested or reported that nonmusicians are more apt than musicians to confuse pitch and timbre when responding to one of those sound properties [9,38,39]. Such confusions would prove particularly challenging when pitch and timbre violate their natural covariance (e.g., accurately labeling the high pitch of a tone when its timbre is dark). This is supported by Lau and colleagues [39], who compared pitch-timbre confusions of infants, adult nonmusicians, and adult musicians. Infants (whose minimal experience with pitch-timbre covariance made them undeterred by violations of it) and musicians (whose extensive experience made them resilient to these violations) suffered far fewer pitch-timbre confusions than nonmusicians (whose intermediate amount of experience rendered them most susceptible to these violations). These examples highlight the second shortcoming of previous research, that musicianship is frequently treated as a binary variable, testing listeners who classify as either (highly experienced) musicians or nonmusicians. While these extreme comparisons might be utilized to maximize the probability of finding a difference between groups, it concurrently undermines the true nature of the variable it is studying. Amount of musical training is a continuous variable, and dichotomizing continuous variables introduces serious statistical challenges in general [40] and in the music perception literature specifically [41]. Further, one’s musical background is a multidimensional construct. Müllensiefen and colleagues [42] have developed a self-report assessment of what they termed ‘musical sophistication’. This questionnaire produces continuous measures of musical training, singing, auditory perceptual abilities, active engagement with music, and emotional responses to music. Using tools such as these, closer connections between musical training and/or background and music perception (and pitch-timbre covariance specifically) can be forged.

Context exists on a multitude of timescales. In many experiments, perceptual performance is subject to short-time perceptual contexts at the level of the experimental session, such as testing order and format. Specifically, while sounds whose pitch-timbre pairings violate their natural covariance are predicted to challenge perception, the extent of this challenge might by modulated by various session-level factors. This challenge could be potentially exacerbated by testing these stimuli at the very beginning of the experiment versus potentially lessened by testing them later in the experiment (once listeners have some experience with the pitches and timbres being tested). This challenge might also be greater with sparser testing of these stimuli and lessened through extended testing. Additionally, overall sensitivity to pitch-timbre pairings might be modulated by blocked testing (completing an entire block of trials where all stimuli either obey or violate the prevailing pattern of covariance) relative to interleaved testing (both trial types are mixed together throughout the test session, more closely modeling how these sounds are encountered in everyday listening). Relevant context also exists on much longer timescales. By adulthood, listeners have had considerable passive exposure to natural covariance between pitch and timbre in both music and in speech. Listeners with musical training have experience above and beyond this through their explicit practice of aural skills, and implicit exposure to a wide range of pitch and timbre combinations in performance and active listening. Measuring how shorter-timescale contexts shape perception and how they interact with longer-timescale contexts such as perceptual expertise is essential for establishing a firm foundation for understanding how perception tracks the natural covariance between musical pitch and timbre.

Here we present results from four experiments that examine perceptual sensitivity to the covariance between musical pitch and timbre. All experiments utilize a pitch labeling task, where each trial presented an instrument playing one tone that listeners labelled as lower pitch (C4) or higher pitch (G4). Exposure and practice with feedback were provided before the main task so that musical training was not a prerequisite for success. Musical background was assessed through the musical training subscale of the Gold-MSI questionnaire [42]. Across all experiments, in accordance with the efficient coding hypothesis, responses were predicted to be faster and more accurate for pitch-timbre pairings that respected their typical covariance (Consistent condition) relative to pairings that violated it (Reversed condition). Performance was also predicted to improve with increased musical training, such that listeners with more extensive musical backgrounds would label pitches more accurately in this task. This outcome would advance the efficient coding hypothesis, demonstrating that sensitivity to statistical structure in the listening environment could be shaped by listener factors such as relevant (musical) perceptual experience.

### Materials and methods

#### Participants.

The study was approved by the Institutional Review Board at the University of Louisville. Participants provided written informed consent and received no financial compensation for their participation.

Target sample sizes for these experiments could not be determined from existing literature, as perceptual sensitivity to pitch-timbre covariance has rarely been investigated directly. Allen and Oxenham [26] measured 20 participants’ ability to discriminate changes in f0 or spectral centroid when the other dimension was varying congruently versus incongruently. Studies that examined sensitivity to pitch-timbre covariance indirectly also had similar or smaller sample sizes [28–30]. McPherson and McDermott [27] tested a larger sample of 105 participants (retaining the data for 86) to “provide evidence for or against the null hypothesis that discrimination was the same for harmonic and inharmonic stimuli”; effects of pitch-timbre congruence were only evaluated via post hoc analysis. Across these studies, results were analyzed at the aggregated level. None of these studies analyzed trial-level data, nor did they analyze responses using mixed-effects regression models.

The appropriate sample size for these experiments was determined in two ways. First, we followed the guidelines of Brysbaert and Stevens [43], who recommended 1600 observations in each condition as a target for properly powered experiments that are being analyzed using mixed-effects regressions. As detailed below, each condition contained 40 trials in an experimental block. Therefore, testing 40 listeners would satisfy this guideline (40 listeners x 40 trials/condition = 1600 observations/condition). Second, we conducted power analyses to estimate target sample sizes. The outcome variable of interest was accuracy, as it more closely aligned with the changes in discriminability as a function of pitch-timbre congruence [23] than response time. Preliminary analyses of listeners’ responses were conducted to inform power analyses, reducing data down to the first two blocks to eliminate potential practice effects (one block apiece of Consistent stimuli and Reversed stimuli as in Experiments 1–3; in Experiment 4 which did not use block structure, all responses were include in analyses). A simplified mixed-effects generalized linear model was constructed analyzing response accuracy with only the fixed effect of condition, random slopes for condition, and random intercepts for listeners. Coefficients from this analysis were extracted to populate a simulated model where sample size was extended to 100 listeners. Power analyses were conducted using the simr [44] package in R, examining the sample size required to yield an acceptable amount of statistical power underlying reliable changes in accuracy across Consistent and Reversed stimuli. The conventional threshold of 80% power for the fixed effect of condition was achieved with sample sizes of 19 (Experiment 1), 19 (Experiment 2), 39 (Experiment 3), and 13 listeners (Experiment 4). This confirmed that testing 40 listeners provided more than sufficient statistical power for the primary outcome variable.

In all, 165 undergraduate students from the Department of Psychological and Brain Sciences at the University of Louisville were tested across four experiments. All reported no known hearing impairments, and received course credit in exchange for their participation. No one participated in multiple experiments. Experiment 1 tested forty undergraduate students (10 men, 28 women, 1 non-binary, 1 not reporting; mean age = 20.79 years, S.D. = 3.47, with 1 not reporting). Experiment 2 tested forty undergraduate students (5 men, 32 women, 1 non-binary, 1 not reporting; mean age = 19.58 years, S.D. = 3.74, with 1 not reporting). Experiment 3 tested forty-one undergraduate students (9 men, 29 women, 3 not reporting; mean age = 20.69 years, S.D. = 5.13, with 1 not reporting). Experiment 4 tested forty-four undergraduate students (three men, 36 women, one non-binary, with four not reporting; mean age = 19.50 years, S.D. = 3.30, with four not reporting).

#### Statistical analyses.

All data were collected from February 19, 2023 to January 30, 2024. Data were analyzed using R version 4.4.0 [45] and the packages *tidyverse* version 2.0.0 [46], *lme4* version 1.1–35.4 [47], *lmerTest* version 3.1−3 [48], and *emmeans* version 1.10.3 [49]. Primary statistical analyses included linear mixed-effects regression models to predict response time on each trial (using the lme4 package) and generalized linear mixed-effects regression models to predict accuracy on each trial (again using the lme4 package). Additional packages that were utilized included *ggplot2* version 3.5.1 [50], *rio* version 1.2.0 [51], *cowplot* version 1.1.3 [52], *ggpubr* version 0.6.0 [53], *gridExtra* version 2.3 [54], *ez* version 4.4−0 [55], and *rstatix* version 0.7.2 [56]. This study’s design and its analysis were not pre-registered. Stimuli, deidentified processed data, and analysis scripts for all main experiments are available in an Open Science Framework repository, https://osf.io/fpj8q/.

#### Stimuli.

**Fig 1:**
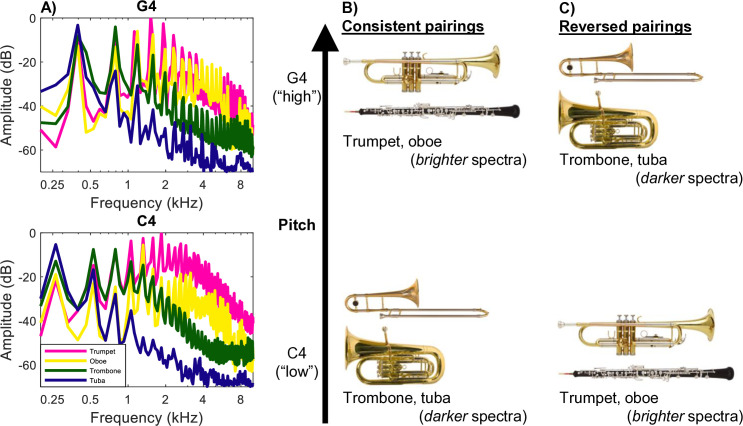
Experimental stimuli and design. (A) Long-term average spectra for the target instruments, arranged in order of decreasing amplitude of high-frequency energy content: trumpet (pink), oboe (yellow), trombone (green), and tuba (blue). Spectra of instruments playing G4 are depicted in the top panel; spectra of instruments playing C4 are depicted in the bottom panel. (B) Pitch-timbre pairings in Consistent blocks: instruments with darker timbres (trombone, tuba) played the lower note (C4), and instruments with brighter timbres (trumpet, oboe) played the higher note (G4). (C) Pitch-timbre pairings in Reversed blocks: instruments with darker timbres played the higher note (G4), and instruments with brighter timbres played the lower note (C4). Musical instrument images courtesy of iStock photos.

Stimuli were recordings of tones played by the trumpet, oboe, trombone, and tuba from the McGill University Master Samples database [57]. These instruments were selected to span a wide range of spectral envelopes (Fig 1a). Recordings of each instrument playing C4 (mean fundamental frequency across the four instruments = 261.33 Hz) and G4 (mean fundamental frequency = 392.14 Hz) were chosen. This large interval was chosen so that notes could be labeled relatively easily by participants with little to no musical training [cf. 11]. These notes occur near the upper end of the registers played by the darker-timbre instruments and toward the lower end of the registers played by the brighter-timbre instruments. Notes were edited to begin at the natural onset of the instrument and terminate at a duration of 1000 ms. A 2-ms linear offset ramp was applied to each recording in MATLAB. Stimuli were then all set to a fixed root mean square amplitude. All experiments used these stimuli.

#### Procedure.

Participants completed the experiment on their personal computers using the online testing platform Gorilla [58]. After the acquisition of informed consent, participants were asked to adjust the computer volume to a comfortable listening level for a sample burst of noise. The full experimental session, which consisted of six steps detailed below, took between 20–30 minutes to complete. All experiments used this same procedure with the exception of the construction of the main task; these are detailed in turn along with each experiment.

First, participants completed a headphone screen to help standardize sound presentation and ensure stimuli were audible. The first screen employed was a tone level discrimination task [59]. On each trial, participants reported which of three tones was the quietest. The correct answer was the tone presented at −6 dB relative to the other two, which is easily distinguishable when listening via headphones. There is also a plausible but incorrect answer of a tone presented 180 degrees out of phase across stereo channels, which sounds quietest over speakers due to destructive interference. Participants who passed the headphone screen had at least five correct responses out of the six trials, within two attempts. Participants who failed the tone level headphone screen twice proceeded to a second headphone screen based on dichotic pitch [60]. In this test, listeners identified which of three noise segments has a faint pitch to it. This pitch, produced by different interaural phase relations in a narrow range of frequencies across the two ears, is detectable over headphones but not over speakers. All participants were retained at this step regardless of whether they passed or failed the headphone screener, since these headphone checks are meant to encourage headphone usage but none of the experimental sounds were dependent upon dichotic sound presentation. Additionally, the authors of these headphone screens acknowledge the possibility of them yielding false negatives [59–60]. Supplementary analyses were conducted after removing responses from listeners who did not pass the headphone screen (n = 5 in Experiment 1, n = 6 in Experiment 2, n = 7 in Experiment 3, n = 5 in Experiment 4). All major patterns of results remained intact; results from these analyses are available in the Open Science Framework repository for this research: https://osf.io/fpj8q/.

Second, participants heard a plucked violin playing the notes C4 and G4 along with the verbal labels of ‘low’ and ‘high’. This was to familiarize participants with the pitches that they were to label as being ‘low’ and ‘high.’ The plucked violin was chosen for presentation in exposure and practice, as this instrument was not heard during the main task. Participants could click a button on the screen to hear these examples up to five times each before proceeding.

Third, participants practiced labeling the plucked violin tones as ‘low’ or ‘high’ pitch 10 times each in random orders (20 total presentations). Participants responded by pressing a corresponding keyboard letter (“e” for low or “i” for high), and feedback was provided on each trial. Participants were required to achieve 90% accuracy in this task and could repeat it two additional times if necessary. Only six participants repeated the practice block, after which point they met the performance criterion. It bears note that instrument timbre was fixed during practice. This was intended to draw listeners’ attention to the pitch dimension, which was essential for their performance in the main experiment. Listeners were not given any instructions or formal training on incongruent pairings of pitch and timbre, as doing so would introduce the risk of artificially inflating performance on those stimuli in the main experiment. At the same time, this methodological decision also presents the risk of poorer performance in the main experiment, whether due to inconsistent responding (e.g., unreliable use of pitch and/or timbre information for making responses) or confusion (e.g., responding to timbre instead of to pitch).

Fourth, the main experiment was patterned after paradigms introduced by Idemaru and Holt [61–62]. On each trial, listeners heard one instrument play one tone. As in practice, participants responded by pressing a keyboard key as quickly and accurately as possible to report whether it was the low tone (C4) or the high tone (G4). The instrument’s pitch and timbre were paired so that they were either congruent with their natural pattern of covariance (Consistent trials: darker timbres of trombone or tuba playing the lower pitch C4, brighter timbres of oboe or trumpet playing the higher pitch G4) or incongruent with this covariance (Reversed trials: darker timbres of trombone or tuba playing the higher pitch G4, brighter timbres of oboe or trumpet playing the lower pitch C4). Most experiments presented stimuli in a blocked manner, where an entire block of 80 trials presented either Consistent sounds or Reversed sounds exclusively (Experiments 1–3; after Idemaru and Holt [61–62]). In Experiment 4, blocks contained both Consistent sounds and Reversed sounds that could occur from trial-to-trial.

Fifth, participants then completed a brief adaptive staircase assessing pitch discrimination abilities [63]. Not all participants successfully completed the staircase task (8 reversals within a maximum of 75 trials). Rather than conduct underpowered analyses due to this data loss, these results are not discussed further.

Sixth and lastly, participants provided responses to the seven-question musical training subscale of the Gold-MSI [42], as well as demographic items (age, gender, hearing health, years of education, income, handedness, native language, tonal language competency, and if they had any technical issues regarding sound presentation).

## Experiment 1

### Design

Experiment 1 was comprised of three blocks (Figs 1b–1c) patterned after paradigms introduced by Idemaru and Holt [61–62]. In the first (Consistent) block, listeners heard instruments playing pitches that respected their natural pattern of covariance. In the second (Reversed) block, listeners heard the same instruments but now playing pitches incongruent to their relative timbre. Finally, the third (Consistent) block was a repetition of the first block. Each block presented 80 trials (four instruments playing their yoked pitch x 20 repetitions). No feedback was provided. Throughout, participants responded by pressing a keyboard key as quickly and as accurately as possible to report whether they heard the low tone or the high tone.

### Results

#### Response time.

As is customary for response time analyses in speeded labeling tasks [4,6,11], only correct responses were retained (removing 1635 trials, or 17.03% of the total data). Additionally, all response times faster than 200 ms were removed, as these responses were too short to reflect the time needed to hear a stimulus and plan a corresponding motor response (e.g., [64]; removing 81 trials, or 1.02% of the remaining data). Distributions of response times were positively skewed, so they were log-transformed to achieve normality. Finally, mean response time was calculated for each participant, and response times exceeding three standard deviations from that listener’s mean were removed (removing 106 trials, or 0.68% of the remaining data).

Linear mixed-effects modeling was used to predict trial-level response times. Fixed effects in this model included block (factor-coded, with the first [Consistent] block serving as the default), Gold-MSI musical training score (mean-centered), and their interaction. The model building process began with a base model with these fixed effects and random intercepts for participants. Random effects were added one at a time and tested via χ^2^ goodness-of-fit tests. If the added term explained significantly more variance, it was retained. This process continued until all random effects of interest were assessed, noting that the final model also had to successfully converge. The final random effects structure included random slopes for block and random intercepts for participants. Model coefficients are listed in Table 1A.

**Table 1:** Mixed-effects modeling results for Experiment 1 (n.b., since models shared fixed effects architectures, results from both models are presented side-by-side for ease of comparison). Results from the linear mixed-effects model analyzing the logarithm of response times are listed at left (A); results from the generalized linear mixed-effects model analyzing response accuracy are listed at right (B). Block 1 (Consistent) was the default level of the factor Block, so all fixed effects are in reference to Block 1.

A) Response Time				Experiment 1	B) Accuracy			
$\hat{\beta }$	SE	*t*	*p*		$\hat{\beta }$	SE	*Z*	*p*
2.919	0.023	127.967	<0.001	Intercept	3.834	0.282	13.607	<0.001
0.011	0.016	0.691	0.490	Block 2	−2.327	0.367	−6.335	<0.001
−0.082	0.018	−4.602	<0.001	Block 3	−0.025	0.339	−0.072	0.942
−0.001	0.002	−0.551	0.582	Gold-MSI	0.053	0.025	2.140	0.032
−0.002	0.002	−1.441	0.151	Block 2 x Gold-MSI	0.114	0.033	3.470	0.001
−0.002	0.002	−1.443	0.161	Block 3 x Gold-MSI	0.047	0.029	1.598	0.110

The first analysis examined block-level context. Relative to the first Consistent block (estimated marginal mean response time, as calculated using the emmeans package [49] = 829.18 ms, SE = 43.54), response times increased numerically but not significantly in the Reversed block (estimated marginal mean response time = 851.10 ms, SE = 48.13), but decreased significantly in the final Consistent block (estimated marginal mean response time = 686.99 ms, SE = 37.40). An additional contrast was tested by recoding the block factor using the Reversed block as the default level and rerunning the model. Mean response times significantly decreased from the Reversed block to the final Consistent block ($\hat{\beta }$ = −0.093, *t* = −5.19, *p* < .0001).

The second analysis examined long-term context. Due to a programming error, three participants did not receive all the questions on the Musical Training subscale of the Gold-MSI [42]. One additional participant completed all tasks through the main experiment but did not complete the Gold-MSI questionnaire. For the remaining 36 participants, responses to these seven-point questions were summed according to the scoring guidelines. Subscale scores can span a range of 7 (lowest musical training score) to 49 (highest musical training score). For Experiment 1, the mean score was 20.80 (SD = 10.87), reflecting a wide range of musical training experience in this listener sample.

**Fig 2:**
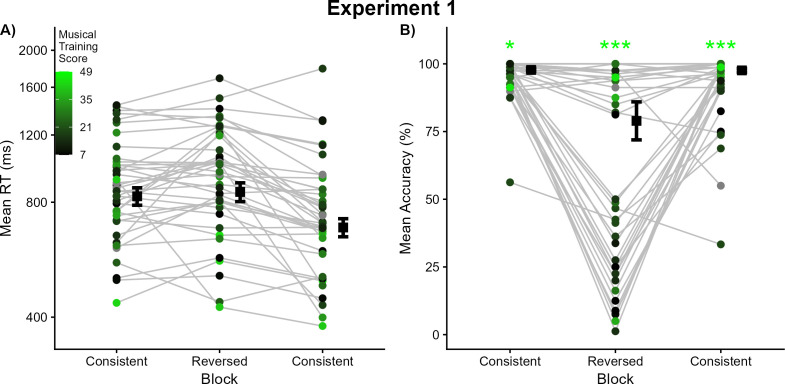
Results from Experiment 1. (A) Each dot represents the mean response time for a given listener in that experimental condition; each listener’s means across conditions are connected by grey lines. The estimated marginal mean response times for each condition are depicted using black squares, with error bars denoting one standard error. Dots are colored according to each listener’s score on the musical training subscale of the Gold-MSI, with brighter (toward green) colors indicating higher scores and darker (toward black) colors indicating lower scores (see inset legend). Grey dots indicate listeners who did not complete the Gold-MSI. (B) Each dot represents the mean accuracy for a given listener in that experimental condition; each listeners’ means across conditions are connected by grey lines, with error bars denoting one standard error. The estimated marginal mean accuracy for each condition is depicted using black squares. Dots are again colored according to each listener’s score on the musical training subscale of the Gold-MSI (the same as in panel A). Asterisks denote a statistically significant influence of musical training scores on performance in that block (*p < .05, **p < .01, ***p < .001).

Gold-MSI scores for each participant are illustrated in Fig 2a, with brighter colors indicating higher scores. These scores were not a significant predictor of trial-level response times in any block (all *t* > −1.62, *p *> .11).

#### Accuracy.

Unlike response time analyses, both correct and incorrect responses were included in accuracy analyses. As above, all response times faster than 200 ms were removed (removing 168 trials, or 1.75% of the remaining data). A generalized linear mixed-effects model was used to predict correct responses on a trial-by-trial basis. Fixed effects in the accuracy model matched those in the response time model detailed above: block (factor-coded, with the first Consistent block serving as the default), Gold-MSI musical training score (mean-centered), and their interaction. The random effects structure was built iteratively as detailed above, arriving at a final structure of random slopes for block and random intercepts for participants. Model coefficients are listed in Table 1B.

The first analysis examined block-level context. Relative to the first Consistent block (estimated marginal mean accuracy = 0.98, SE = 0.01), accuracy decreased sharply in the Reversed block (estimated marginal mean accuracy = 0.79, SE = 0.07) but did not differ across the first and final Consistent blocks (estimated marginal mean accuracy = 0.98, SE = 0.01). Additional contrasts were tested by recoding the block factor using the Reversed block as the default level and rerunning the model. Accuracy significantly increased from the Reversed block to the final Consistent block ($\hat{\beta }$ = 2.30, *Z* = 5.04, *p* < .0001).

The second analysis examined longer-term context. Gold-MSI scores for each participant are illustrated in Fig 2b, with brighter colors indicating higher scores on the musical training subscale. These scores were significant predictors of response accuracy in each block, with higher musical training scores corresponding to more accurate performance (first Consistent block: $\hat{\beta }$ = 0.05, *Z* = 2.14, *p* < .05; Reversed block: $\hat{\beta }$ = 0.17, *Z* = 4.16, *p* < .0001; final Consistent block: $\hat{\beta }$ = 0.10, *Z* = 3.55, *p* < .001). Interactions between block and Gold-MSI scores indicate that this relationship contributed significantly more to accuracy in the Reversed block than the first Consistent block.

#### Discussion.

Listeners exhibited clear perceptual sensitivity to typical patterns of covariation between musical pitch and timbre. This finding coheres with the efficient coding hypothesis [18–19], as performance indicates listeners have learned this prevailing pattern of covariation in the acoustic environment and that performance worsens when sounds violate this relationship. Importantly, these patterns of performance were correlated with listeners’ musical training. This marks an important advance for the efficient coding hypothesis, as its previous applications to auditory perception frequently treated sensitivity to statistical structure in the sensory environment as uniform. Here, this sensitivity was related to listener factors such as relevant perceptual experience. Listeners reporting more musical training might have had more experience hearing reversed pairings of pitch and timbre (and thus performed more accurately on these trials) than listeners reporting less musical training (and thus performed less accurately on these trials). While the relationship between musical training and task performance is correlational and not causal, it nevertheless highlights graded sensitivity to stimulus statistical structure across a listener sample. These points are revisited in the General Discussion.

Perceptual sensitivity to pitch-timbre covariance was affected by context both at the immediate level across blocks and linked to the longer context of participants’ musical training. Here we unify the response time and accuracy results and discuss the shorter block context followed by the longer experience context. At the block level, the first (Consistent) block established baseline levels of performance in terms of mean response time and (very high) mean accuracy. Despite having one block’s worth of experience in the task, in the second block, accuracy dropped sharply and response times increased numerically but not significantly. This indicates the challenge of labeling pitches when timbre is varying in a manner that violates their covariance (but, as the next paragraph will discuss, this challenge was not equal for all participants). These results build upon previous studies of pitch-timbre interference [7–10,15]: timbre varied throughout the pitch labeling task, but only when timbre varied in contrast with its typical covariance with pitch was perception challenged. Finally, when stimuli returned to their common pairings of pitch and timbre in the final block, response times were fastest overall and accuracy returned to ceiling levels.

The longer timescale of perceptual context considered was the perceptual expertise from musical training. Numerous studies have demonstrated superior pitch perception for listeners with considerable musical training as compared to listeners without musical training (e.g., [65–67]). Here, listeners were recruited irrespective of their musical backgrounds, which varied continuously as assessed by the musical training subscale of the Gold-MSI [42]. Gold-MSI scores were not predictive of response times, but were significant predictors of accuracy in every block, with a stronger association in the Reversed block than in the first Consistent block. Gold-MSI scores also shed light on the mean accuracies in the Reversed block being broadly split into two groups: higher performance (>80%) or poorer performance (≤50%; Fig 2b). Many of the participants with more musical training performed better in this block (mean Gold-MSI score for listeners in the higher-performing group = 26.67) whereas many of the participants with less-to-no musical training performed more poorly (mean Gold-MSI score for listeners in the poorer-performing group = 15.00; Welch’s two-sample *t*-*t*est: *t*_28.71 _= 3.78, *p* < .001). This finding suppor*t*s previous results where listeners without musical training were more apt to confuse pitch and timbre than listeners with musical training [9,38,39]. While inconsistent use of pitch versus brightness information in responses would result in near-chance accuracy (50%), several participants performed markedly below chance, which can only be achieved by labeling the timbre of each note as low or high (when it was in fact dark or bright) rather than its pitch, as instructed. This confusion was not due to an inability to perform the task, as Fig 2b illustrates good-to-excellent performance for these participants in the Consistent blocks preceding and following the Reversed block. The significant influences of Gold-MSI scores on accuracy in the Consistent blocks suggest that the benefits of musical training are relatively global for this task, and not limited to cases where pitch and timbre are heard in combinations that violate their typical covariance.

In addition to adopting its experimental paradigm, the present results bear other similarities to dimension-based statistical learning [61,62,68]. In those studies, listeners categorized speech sounds based on whether two acoustic cues were presented in their typical (canonical) or atypical (reversed) combinations. Both in dimension-based statistical learning and here, listeners’ responses were primarily reliant on a dominant acoustic property within the pattern of covariance (here, pitch, as it was highly germane to the pitch labeling task) while also exhibiting sensitivity to a secondary property (timbre). Listeners exhibited rapid perceptual adjustments when the relationship between stimulus properties changed (moving from the Consistent block to the Reversed block or vice versa). But, key differences across paradigms must also be noted. Listeners produced objectively correct or incorrect responses in the pitch labeling task whereas dimension-based statistical learning assesses categorization where accuracy does not necessarily apply, instead measuring perceptual cue weights or how often a particular response was provided. Second, while both paradigms involve presenting stimuli with covarying stimulus dimensions, the relationship between the dimensions differ. Here, pitch is essential to the perceptual task and timbre variation is technically irrelevant; in dimension-based statistical learning, the acoustic redundancy of speech results in either acoustic dimension being sufficient and neither being necessary for producing a response.

While poorer performance in the Reversed block is being attributed to those stimuli violating typical patterns of pitch-timbre covariance, an alternative explanation is possible. Rather than following natural signal statistics, perception could have followed a simpler pattern of superior performance on stimuli heard in the first block, then poorer performance on new stimuli heard in the second block (when pitch-timbre pairings were reversed). One way to disentangle these interpretations is to reverse the order of testing blocks, as has been done in studies of dimension-based statistical learning [61,62,68]. If perception is following the natural stimulus statistics of pitch-timbre covariance, then patterns of performance should also invert, being poorer in the first and final blocks of the experiment (Reversed) relative to the second block (Consistent). If perception is instead following low-level stimulus order effects, then the pattern of results in Experiment 1 should be replicated (good performance followed by poor performance then a return to good performance). This was one of the principal motivations behind conducting Experiment 2.

A second motivation for inverting the order of test blocks was to examine performance on a new timescale of perceptual context. Experiment 2 maintains the same opportunities to examine perception on block-level and long-term timescales of context while also examining performance at the level of the experimental session more broadly. In Experiment 1, perception of Reversed stimuli in the second block was influenced by not only their (violation of) stimulus statistics, but also relative to having labeled Consistent stimuli in the first block. Testing Consistent and Reversed blocks in a different order provides an examination of how performance varies as a function of session context (that is, by being tested first and not having heard any other stimuli yet versus by being tested second and being influenced by having just heard the first block of stimuli as in Experiment 1).

In Experiment 2, block order was changed such that Reversed stimuli were tested in the first and third blocks while Consistent stimuli were tested in the second block only. Similar to Experiment 1, the base prediction of superior pitch labeling for Consistent stimuli still holds. Given that the amount of testing with Reversed stimuli has doubled, performance on these trials is predicted to significantly improve in the third block relative to the first block. Two possibilities exist for how performance with Reversed stimuli in Experiment 2 will compare to that of Experiment 1. If Experiment 1 performance was buoyed by task practice effects, then performance in the first Reversed block of Experiment 2 will be comparatively poorer. Conversely, if testing Consistent stimuli first in Experiment 1 primed listeners on typical pitch-timbre covariance and thus suppressed their performance with Reversed stimuli in the second block, then performance in the first Reversed block of Experiment 2 will improve. As both possibilities are plausible, the results of Experiment 2 will reveal which has more explanatory power.

## Experiment 2

### Procedure

The procedure matched that of Experiment 1 but with one change. In Experiment 1, blocks in the main task were tested in the order Consistent – Reversed – Consistent. In Experiment 2, this order was inverted, testing blocks in the order Reversed – Consistent – Reversed.

### Results

#### Response time.

As in Experiment 1, only correct responses were retained (removing 2683 trials, or 28.67% of the total data) and all response times faster than 200 ms were removed (removing 88 trials, or 1.32% of the remaining data). Distributions of response times were positively skewed, so they were log-transformed to achieve normality. Finally, all responses exceeding three times the standard deviation of each listener’s remaining mean response times were removed (removing 88 trials, or 0.67% of the remaining data). After these steps, all results from one participant were removed owing to an abnormally long response times in the first testing block (mean = 5100 ms), leaving a final sample of 39.

Linear mixed-effects modeling was used to predict trial-level response times using the same fixed effects as described in Experiment 1: block (factor-coded, with the first Reversed block serving as the default), Gold-MSI musical training score (mean-centered), and their interaction. The random effects structure was built iteratively following the same procedures outlined in Experiment 1. The final random effects structure included random slopes for block and random intercepts for participants. Model coefficients are listed in Table 2A.

**Table 2:** Mixed-effects modeling results for Experiment 2 (n.b., since models shared fixed effects architectures, results from both models are presented side-by-side for ease of comparison). Results from the linear mixed-effects model analyzing the logarithm of response times are listed at left; results from the generalized linear mixed-effects model analyzing response accuracy are listed at right. Block 1 (Reversed) was the default level of the factor Block, so all fixed effects either depict or are in reference to Block 1.

A) Response Time				Experiment 2	B) Accuracy			
$\hat{\beta }$	SE	T	p		$\hat{\beta }$	SE	*Z*	*p*
2.983	0.022	137.600	<0.001	Intercept	1.231	0.389	3.164	0.002
−0.087	0.016	−5.571	<0.001	Block 2	2.356	0.345	6.834	<0.001
−0.075	0.015	−4.888	<0.001	Block 3	0.105	0.187	0.562	0.574
−0.004	0.002	−1.715	0.096	Gold-MSI	0.148	0.040	3.731	<0.001
0.002	0.002	1.576	0.125	Block 2 x Gold-MSI	−0.085	0.035	−2.473	0.013
−0.000	0.002	−0.198	0.844	Block 3 x Gold-MSI	−0.017	0.019	−0.922	0.356

The first analysis examined block-level context. Relative to the first Reversed block (estimated marginal mean response time = 962.46 ms, SE = 48.05), response times were significantly faster in the Consistent block (estimated marginal mean response time = 786.92 ms, SE = 31.18) and in the final Reversed block (estimated marginal mean response time = 810.50 ms, SE = 38.49). An additional contrast was tested by recoding the block factor using the Consistent block as the default level and rerunning the model. Response times did not significantly change from the Consistent block to the final Reversed block ($\hat{\beta }$ = 0.013, *t* = 0.87, *p* = .39).

The second analysis examined session-level context. Response times could also vary at the experimental session level depending on whether Consistent trials were tested first followed by Reversed trials (Experiment 1) or Reversed followed by Consistent (Experiment 2). To explore this timescale, data were reduced to the first two blocks of Experiments 1 and 2 (one block apiece of Consistent and Reversed pairings, which both experiments shared) and analyzed in mixed-effect models with fixed main effects of Block, Gold-MSI musical training scores, and Experiment, and all interactions between these terms. Block was categorically coded according to the contents of each block (Consistent or Reversed stimuli) to examine how their perception varied as a function of different testing orders. In these analyses, fixed effects and interactions involving Experiment were of primary interest.

Mean response times did not differ across samples for either Consistent stimuli or for Reversed stimuli (main effects of Experiment: *t *< 1.65, *p *> .10). However, the experimental session altered the pattern of mean response times (Condition-by-Experiment interaction: $\hat{\beta }$ = 0.076, *t* = 3.35, *p* < .01). This interaction reflects the fact *t*hat mean response times did not significantly differ between the first and second blocks in Experiment 1 (829 ms in the [first] Consistent block, 851 ms in the [second] Reversed block) but they did significantly differ in Experiment 2 (787 ms in the [second] Consistent block, 962 ms in the [first] Reversed block).

The third analysis examined long-term context. Thirty-nine participants completed the Gold-MSI musical training subscale. The mean score (21.32, SD = 9.97) was highly comparable to that of Experiment 1 participants (independent-samples *t*-test: *t*_73_ = 0.25, *p* = 0.80; see S1 Fig for distributions of Gold-MSI scores for each listener sample). Gold-MSI scores for each participant are illustrated in Fig 2 with brighter colors indicating higher scores. These scores trended toward being negative predictors of response times in the Reversed blocks (first Reversed block: $\hat{\beta }$ = −0.004, *Z* = −1.72, *p* = .10; final Reversed block: $\hat{\beta }$ = −0.004, *Z* = −1.95, *p* = .06), but showed no association with response times in the Consistent block ($\hat{\beta }$ = −0.001, *Z* = −0.73, *p* = .47). There was also a trend toward stronger association between musical training scores and response times in the final Reversed block than the Consistent block ($\hat{\beta }$ = −0.003, *Z* = −1.88, *p* = .07), but caution is warranted interpreting this result given that musical training itself was not a significant predictor of response times in either block.

#### Accuracy.

As in Experiment 1, both correct and incorrect responses were included in accuracy analyses, and all response times faster than 200 ms were removed (removing 179 trials, or 1.86% of the remaining data). While response times were not being analyzed here, results from the one participant with abnormally long response times in the first testing block were removed from accuracy analyses as well. A generalized linear mixed-effects model was used to predict correct responses on a trial-by-trial basis. Fixed effects in the accuracy model matched those in the response time model detailed above: Block (factor-coded, with the first Reversed block serving as the default), Gold-MSI musical training score (mean-centered), and their interaction. The random effects structure was built iteratively as detailed above, arriving at a final structure of random slopes for block and random intercepts for Participants. Model coefficients are listed in Table 2B.

The first analysis examined block-level context. Relative to the first Reversed block (estimated marginal mean accuracy = 0.76, SE = 0.07), accuracy increased sharply in the Consistent block (estimated marginal mean accuracy = 0.97, SE = 0.01), then returned to a lower level in the final Reversed block (estimated marginal mean accuracy = 0.78, SE = 0.07). An additional contrast was tested by recoding the block factor using the Consistent block as the default level and rerunning the model. Accuracy significantly decreased from the Consistent block to the final Reversed block ($\hat{\beta }$ = −2.25, *Z* = −6.36, *p* < .0001).

The second analysis examined session-level context. Potential changes in accuracy across Experiments 1 and 2 as a function of the order of testing blocks were also assessed. As in the analyses of response times, data were reduced to the first two blocks of Experiments 1 and 2 and analyzed in mixed-effect models with fixed main effects of Block, Gold-MSI musical training score, and Experiment, and all interactions between these terms. Block was again categorically coded according to the contents of each block (Consistent or Reversed stimuli). Base levels of performance did not differ across experiments (fixed effects of Experiment with either Consistent or Reversed as the default level of condition: *Z* > −1.01, *p* > .31), nor did the difference in accuracy across blocks (Condition-by-Experiment interaction: $\hat{\beta }$ = 0.09, *Z* = 0.18, *p* = .86).

**Fig 3:**
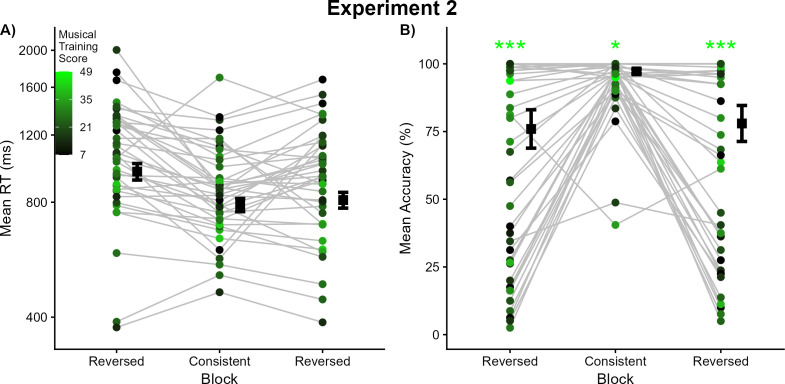
Results from Experiment 2. (A) Each dot represents the mean response time for a given listener in that experimental condition; each listener’s means across conditions are connected by grey lines. The estimated marginal mean response times for each condition are depicted using black squares, with error bars denoting one standard error. Dots are colored according to each listener’s score on the musical training subscale of the Gold-MSI, with brighter (toward green) colors indicating higher scores and darker (toward black) colors indicating lower scores (see inset legend). (B) Each dot represents the mean accuracy for a given listener in that experimental condition; each listeners’ means across conditions are connected by grey lines. The estimated marginal mean accuracy for each condition is depicted using black squares, with error bars denoting one standard error. Dots are again colored according to each listener’s score on the musical training subscale of the Gold-MSI (the same as in panel A). Asterisks denote a statistically significant influence of musical training scores on performance in that block (*p < .05, **p < .01, ***p < .001).

The third analysis examined long-term context. Gold-MSI scores for each participant are illustrated in Fig 3b, with brighter colors indicating higher scores on the musical training subscale. These scores were again significant predictors of response accuracy in each block, with higher musical training scores corresponding to more accurate performance (first Reversed block: $\hat{\beta }$ = 0.15, *Z* = 3.73, *p* < .001; Consistent block: $\hat{\beta }$ = 0.06, *Z* = 2.09, *p* < .05; final Reversed block: $\hat{\beta }$ = 0.13, *Z* = 3.30, *p* < .001). Interactions between block and Gold-MSI scores indicate that musical training contributed significantly more to accuracy in the first Reversed block than the Consistent block ($\hat{\beta }$ = 0.09, *Z* = 2.47, *p* < .05) and trended in that direction for the final Reversed block as well ($\hat{\beta }$ = 0.07, *Z* = 1.92, *p* = .055).

### Discussion

The results of Experiment 1 could be explained by patterns of covariance between musical pitch and timbre (superior performance in Consistent blocks relative to the Reversed block) or by low-level session effects (superior performance in the first block relative to the second block, which featured new pitch-timbre pairings). Experiment 2 disentangled these interpretations by reversing the order of testing blocks. Performance in both experiments follows patterns of covariance between pitch and timbre: superior performance in Consistent blocks relative to the Reversed blocks in whichever order they were tested. This parallels reports of perceptual plasticity in studies of dimension-based statistical learning [61,62,68], where perception tracked the stimulus statistics in whichever order the blocks were presented.

Inverting the order of test blocks in Experiment 2 resulted in a clear inversion of block-level performance. When the Reversed block was tested first, responses were still slower and less accurate than responses in the Consistent block, as predicted. With the amount of testing with Reversed stimuli doubling, performance on these trials was predicted to significantly improve in the third block. This prediction was only partially supported, as responses were significantly faster than but equally accurate to that of the first Reversed block. This decrease in response times indicates a practice effect, but it was not accompanied by an increase in accuracy, even with considerable room for improvement being available (estimated marginal mean accuracy = 0.78). This finding is further discussed below.

Session-level analyses revealed how performance changed as a function of block testing order across Experiments 1 and 2. While response times did not significantly differ when Consistent stimuli were tested first and Reversed stimuli were tested second (an increase of 39 ms across blocks in Experiment 1), a sharp decrease in response times was observed when Reversed stimuli were tested first and Consistent stimuli second (a decrease of 185 ms across blocks in Experiment 2). This pattern of results cannot be attributed to differences in listener samples, as response time and accuracy performance were well-matched across experiments in the Consistent block and in the Reversed block. Thus, labeling the pitches of Reversed stimuli in Experiment 1 appears to be aided by the practice effects of having completed a Consistent block first; when Reversed stimuli were tested first instead, responses became significantly slower.

Musical training scores on the Gold-MSI were again predictive of accuracy in every block, but were not predictive of response time in any block (although nonsignificant trends were observed in the Reversed blocks). As was also observed in Experiment 1, musical training was more strongly associated with accuracy on Reversed trials than Consistent trials (among the first two blocks of each experiment). Thus far, the long-term context of musical training continually bears on how accurately listeners labeled pitches when timbre is varying, but not on how quickly these responses were provided. As in Experiment 1, this portends a broad benefit of musical training for this pitch labeling task, however the instrument timbre is paired with it.

Relative to the first Reversed block, response times decreased but accuracy did not increase when Reversed stimuli were tested again in the third block. This same pattern of results moving from the first to the third block occurred in Experiment 1 as well, but accuracy was already at ceiling levels for Consistent stimuli so there was no room for improvement in the final testing block (estimated marginal mean accuracies = 0.98 in each Consistent block). Even with twice the number of trials, listeners in Experiment 2 did not outperform listeners in Experiment 1 on Reversed trials (estimated marginal mean accuracies spanned 0.76–0.79). This might indicate saturation effects where performance by listeners with mixed musical backgrounds improves no further. The reason underlying this saturation effect is unclear. The issue does not lie with the pitches nor instruments selected, as those are very well recognized and labeled in the Consistent block (Figs 2b and 3b). This also cannot be attributed to musical background, as the predictive power of Gold-MSI scores for Reversed trial accuracy did not change across experiments ($\hat{\beta }$ = 0.003, *Z* = 0.08, *p* = .94).

While one cannot expect the highest-performing listeners to improve upon their already ceiling levels of performance with Reversed stimuli, lower-performing listeners have considerable room for improvement. Accuracy results in the Reversed blocks of Experiment 2 varied more continuously than in Experiment 1, where they were bifurcated into higher- and lower-performers (Fig 2b). In both experiments, near-chance performance reflects inconsistent use of pitch and brightness information in responses, and below-chance performance reflects confusion by labeling the brightness of the tone instead of its pitch. Whether listeners are aware that they are making these mistakes in real time is unclear, as the experimental design proceeded identically whether responses were correct or incorrect. If listeners received feedback about the accuracy of their responses, that would provide a means to recognize they were making mistakes and change their response strategies to improve performance. As extended testing without feedback failed to improve Reversed accuracy in Experiment 2, adding trial-by-trial feedback is likely to lead to a more immediate improvement in performance, even inside of a single testing block.

Trial-by-trial feedback is predicted to improve performance in the Reversed block, where accuracy is lower overall and seems to saturate. To test this prediction, Experiment 3 adopted the procedure of Experiment 1 but added trial-by-trial feedback following each response. Given reliable performance levels established for Reversed trials without feedback (estimated marginal means = 0.76–0.79), accuracy for Reversed trials with feedback is predicted to surpass these levels. Given that performance with Consistent stimuli was already at/near ceiling levels without feedback, the introduction of feedback is not predicted to have any influence in this condition. Additionally, comparisons across experiments with and without feedback (Experiments 3 and 1, respectively) provides an additional examination of session-level context where stimuli and their testing block orders remain fixed but other experimental factors varied. Concomitant with the predicted increase in accuracy, responses to Reversed stimuli are predicted to be faster with feedback than without it.

## Experiment 3

### Procedure

The procedure matched that of Experiment 1 (Consistent-Reversed-Consistent Blocks) with one change. At the end of each trial, as soon as the listener registered their response, they received immediate feedback regarding the accuracy of their response (a green checkmark for a correct response, a red X for an incorrect response).

### Results

#### Response time.

As in other experiments, only correct responses were retained (removing 489 trials, or 4.97% of the total data) and all response times faster than 200 ms were removed (removing 27 trials, or 0.29% of the remaining data). Distributions of response times were positively skewed, so they were log-transformed to achieve normality. Finally, all responses exceeding three times the standard deviation of each listener’s mean response time of the remaining trials were removed (removing 121 trials, or 0.65% of the remaining data).

Linear mixed-effects modeling was used to predict trial-level response times using the same fixed effects as described in previous experiments: Trial number (mean-centered), Block (factor-coded, with the first Consistent block serving as the default), and their interaction. The random effects structure was built iteratively following the same procedures outlined previously. The final random effects structure included random slopes for Block and random intercepts for Participants. Model coefficients are listed in Table 3A.

**Table 3:** Mixed-effects modeling results for Experiment 3 (n.b., since models shared fixed effects architectures, results from both models are presented side-by-side for ease of comparison). Results from the linear mixed-effects model analyzing the logarithm of response times are listed at left; results from the generalized linear mixed-effects model analyzing response accuracy are listed at right. Block 1 (Consistent) was the default level of the factor Block, so all fixed effects either depict or are in reference to Block 1.

A) Response Time				Experiment 3	B) Accuracy			
$\hat{\beta }$	SE	t	p		$\hat{\beta }$	SE	*Z*	*p*
2.885	0.021	136.944	<0.001	Intercept	3.825	0.165	23.154	<0.001
−0.014	0.014	−1.045	0.308	Block 2	−1.005	0.134	−7.520	<0.001
−0.056	0.015	−3.847	0.015	Block 3	−0.391	0.144	−2.707	0.007
−0.004	0.002	−1.715	0.098	Gold-MSI	0.024	0.016	1.555	0.120
−0.001	0.001	−0.993	0.332	Block 2 x Gold-MSI	0.041	0.012	3.269	0.001
−0.001	0.001	−0.387	0.716	Block 3 x Gold-MSI	−0.011	0.013	−0.818	0.413

The first analysis examined block-level context. Relative to the first Consistent block (estimated marginal mean response time = 766.89 ms, SE = 37.20), response times were not significantly different in the Reversed block (estimated marginal mean response time = 741.89 ms, SE = 38.20) but were significantly faster in the final Consistent block (estimated marginal mean response time = 674.44 ms, SE = 32.18). An additional contrast was tested by recoding the block factor using the Reversed block as the default level and rerunning the model. Response times also significantly decreased from the Reversed block to the final Consistent block ($\hat{\beta }$ = −0.041, *t* = −3.77, *p* < .001).

The second analysis examined session-level context. Response times could also vary at the experimental session level depending on whether feedback was present (Experiment 3) or absent (Experiment 1). To explore this context timescale, responses across both experiments were analyzed in mixed-effect models with fixed main effects of Block, Gold-MSI musical training scores, and Experiment, and all interactions between these terms. As in previous analyses, Block and Experiment were both categorically coded. This was facilitated by the same conditions being tested in the same order in both experiments. Fixed effects and interactions involving experiment were of primary interest.

Response times were numerically but not significantly faster with feedback for the Consistent condition (main effect of Experiment: *t *= −1.09, *p* = .28) and the Reversed condition (main effect of Experiment: *t *= −1.79, *p* = .08). The only session-level influence on response times was the difference in response times in the Reversed and final Consistent block being reduced in Experiment 3 (Block-by-Experiment interaction: $\hat{\beta }$ = 0.05, *t* = 2.51, *p* < .05). Without feedback in Experiment 1, mean response *t*ime decreased by 164 ms from the Reversed block to the final Consistent block; with feedback in Experiment 3, this decrease was only 67 ms.

**Fig 4:**
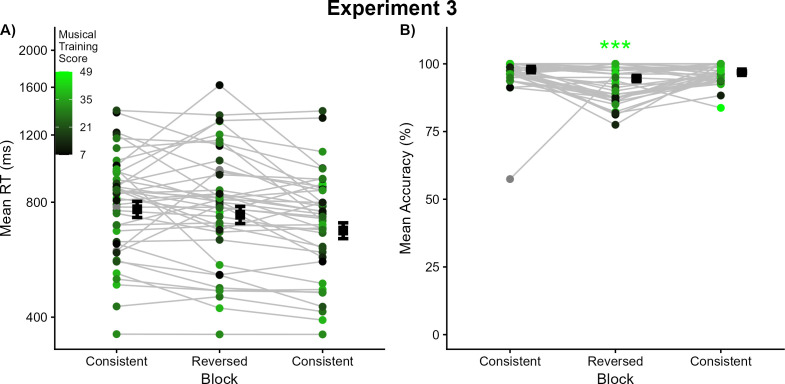
Results from Experiment 3. (A) Each dot represents the mean response time for a given listener in that experimental condition; each listener’s means across conditions are connected by grey lines. The estimated marginal mean response times for each condition are depicted using black squares, with error bars denoting one standard error. Dots are colored according to each listener’s score on the musical training subscale of the Gold-MSI, with brighter (toward green) colors indicating higher scores and darker (toward black) colors indicating lower scores (see inset legend). Grey dots indicate listeners who did not complete the Gold-MSI questions. (B) Each dot represents the mean accuracy for a given listener in that experimental condition; each listeners’ means across conditions are connected by grey lines. The estimated marginal mean accuracy for each condition is depicted using black squares, with error bars denoting one standard error. Dots are again colored according to each listener’s score on the musical training subscale of the Gold-MSI (the same as in panel A). Asterisks denote a statistically significant influence of musical training scores on performance in that block (* *p* < .05, ** *p* < .01, *** *p* < .001).

The third analysis examined long-term-level context. One participant did not continue after completing the main experiment but before completing the Gold-MSI questionnaire; another participant completed only a portion of the survey. The remaining 39 participants completed the Gold-MSI musical training subscale. The mean score (22.62; SD = 10.25) was again highly comparable to that in previous experiments (independent-samples *t*-test against Experiment 1 participants: *t*(73) = 0.73, *p* = 0.47). Gold-MSI scores for each participant are illustrated in Fig 4a with brighter colors indicating higher scores. These scores were significant predictors of response times in the Reversed block, with higher scores corresponding to faster response times ($\hat{\beta }$ = −0.005, *Z* = −2.23, *p* < .05). Gold-MSI scores trended in the same direction in both Consistent blocks (first Consistent block: $\hat{\beta }$ = −0.004, *Z* = −1.72, *p* = .09; final Consistent block: $\hat{\beta }$ = −0.004, *Z* = −2.01, *p* = .051). None of the interactions between block and Gold-MSI scores reached statistical significance (all $\hat{\beta }$ < 0.002, *Z* < 1.00, *p* > .32).

#### Accuracy.

All response times faster than 200 ms were removed (removing 49 trials, or 0.50% of the data). A generalized linear mixed-effects model was used to predict correct responses on a trial-by-trial basis. Fixed effects in the accuracy model matched those in the response time model detailed above: Block (factor-coded), Gold-MSI musical training scores (mean-centered, with the first Consistent block serving as the default), and their interaction. The random effects structure was built iteratively as detailed above, but adding random slopes for Block did not significantly improve model fit (*p* = .076). Therefore, the final model structure included only random intercepts for participants. Model coefficients are listed in Table 3B.

The first analysis examined block-level context. Relative to the first Consistent block (estimated marginal mean accuracy = 0.98, SE = 0.003), accuracy decreased significantly in the Reversed block (estimated marginal mean accuracy = 0.95, SE = 0.007), and decreased slightly but significantly in the final Consistent block as well (estimated marginal mean accuracy = 0.97, SE = 0.005). An additional contrast was tested by recoding the block factor using the Reversed block as the default level and rerunning the model. Accuracy increased from the Reversed block to the final Consistent block ($\hat{\beta }$ = 0.61, *Z* = 5.15, *p* < .0001).

The second analysis examined session-level context. Session-level effects were analyzed using mixed-effects models with the same architecture detailed above for response time analyses. With the first Consistent block as the default level of block, only one interaction with experiment reached statistical significance; for ease it will be described below. When the Reversed block was set to the default level, feedback dramatically improved accuracy in the Reversed block in Experiment 3 relative to Experiment 1 (fixed effect of Experiment: $\hat{\beta }$ = 1.82, *Z* = 4.124, *p* < .001). Due to this improvement, the gaps in performance between Reversed and neighboring blocks in Experiment 1 (differences of 0.18–0.19) also significantly decreased in Experiment 3 (differences of 0.02–0.03; Condition-by-Experiment interactions: $\hat{\beta }$ <−1.60, *Z* < −4.23, *p* < .0001).

The third analysis examined long-term context. Gold-MSI musical training scores for each participant are illustrated in Fig 4b, with brighter colors indicating higher scores on the musical training subscale. These scores had a positive influence on accuracy in the Reversed block ($\hat{\beta }$ = 0.07, *Z* = 4.75, *p* < .0001). Unlike other experiments, no such associations were evident in either Consistent block ($\hat{\beta }$ < 0.03, *Z* < 1.56, *p* > .12). As a result, the relationship between Gold-MSI scores and accuracy was significantly stronger in the Reversed block than in either Consistent block (Block-by-Gold-MSI interactions: $\hat{\beta }$ > 0.04, *Z* > 3.26, *p* < .005).

While musical training scores were significant predictors of response accuracy in the Reversed block, the introduction of trial-by-trial feedback obscures this relationship somewhat. If the addition of feedback resulted in musical background only briefly influencing response accuracy at the beginning of the block, this would seriously challenge there being a true relationship between this long-term context and task performance. Conversely, if musical background has an enduring influence on response accuracy throughout the block with its trial-by-trial feedback, that would reinforce the relationship between long-term context and task performance. Data were split into the first 40 trials and the second 40 trials within each block and reanalyzed. All patterns of results remained intact: musical training did not contribute to response accuracy in either half of the Consistent blocks, but it was a significant contributor to task performance in both halves of the Reversed block, confirming their close relationship. These analyses are available in the Open Science Framework repository for this research: https://osf.io/fpj8q/.

### Discussion

Experiments 1 and 2 varied the order and amount of testing on stimuli that violated typical patterns of pitch-timbre covariance, but mean accuracies for these stimuli plateaued around 80% correct. No feedback had been provided about response accuracy, so this saturation might have been due in part to listeners being unaware when they made mistakes (e.g., labeling the brightness of the tone instead of its pitch). Experiment 3 added trial-by-trial feedback about response accuracy which was predicted to improve this performance. This improvement was apparent on both shorter-term (block-level) and longer-term (musical background) timescales of perceptual context.

Results again followed the overall patterns of past experiments, with superior pitch labeling when pitch and timbre respected their typical covariance rather than violated it. However, in the presence of feedback, this decrease in performance was extremely modest (estimated marginal means: the first Consistent block = 0.98, the Reversed block = 0.95). Performance in the Reversed block was sufficiently high such that the improvement in mean accuracy for the final Consistent block was extremely modest (estimated marginal mean = 0.97). Feedback did not appear to alter patterns of response times, which instead more closely resembled general practice effects (slower response times in the first two blocks relative to the final block).

Most importantly, labeling of Reversed stimuli dramatically improved relative to when no feedback was provided (estimated marginal means rising from 0.82 in Experiment 1 to 0.95 in Experiment 3). Feedback provided listeners with an opportunity to recognize when they were confusing pitch with timbre, correcting their mistakes and improving their pitch labeling for Reversed stimuli. Conversely, feedback did not improve accuracy in the two Consistent blocks because it was already at ceiling levels (estimated marginal means of 0.97–0.98 across Experiments 1 and 3), nor did it significantly decrease response times. Results confirmed all predictions regarding the addition of feedback, lessening the perceptual challenge posed by Reversed stimuli but not affecting the (already ceiling-level) performance with Consistent stimuli.

Feedback also affected the relationship between task performance and musical training. In Experiments 1 and 2, musical training scores from the Gold-MSI were repeatedly predictive of accuracy in every experimental block. As predicted, feedback improved accuracy in the Reversed block of Experiment 3, and this significant relationship maintained. Surprisingly, unlike previous studies, mean accuracy in the Consistent blocks was no longer correlated with musical training scores. This was not due to differences in musical background across Experiments 1 and 3 (comparable Gold-MSI scores) nor baseline performance (comparable mean accuracies in Consistent blocks across experiments). Instead, the dissolution of this relationship was due in part to the improvement by listeners with less musical training. In the strong associations between accuracy and Gold-MSI scores observed previously, the less musically trained listeners had been likely to contribute poorer performance relative to the rest of the sample (and more musically trained listeners tended to perform more accurately; e.g., see Experiment 1 discussion for analyses). With feedback, less-musically-trained listeners improved to the point where the relationship between accuracy and musical training scores was broken.

Listeners of all musical training backgrounds have had considerable exposure to typical patterns of pitch-timbre covariance before participating in any of the reported experiments. This is evident from their performance immediately reflecting this stimulus regularity: near/at ceiling-level performance in the first blocks of Experiments 1 and 3 and weaker performance in the first block on Experiment 2. Yet, all experiments thus far have utilized blocked stimulus presentation. In whichever order blocks were presented, each contained 80 trials of the same experimental condition (Consistent or Reversed). While the pitches and timbres could change from trial to trial, the statistical relationship between them was held constant throughout that block. While methodologically expedient, blocked testing is a marked departure from how listeners encounter these sounds in everyday listening. Listeners hear wide ranges of pitches and timbres, with some offering strong evidence for or against their typical pattern of covariance and others offering weak evidence either way (e.g., pitches not readily labeled as low or high given the musical context, timbres with brightnesses between these bright and dark extremes). Importantly, listeners are rarely if ever in situations where brighter-timbre instruments are playing only higher pitches and darker-timbre instruments are playing only lower pitches (or vice versa) for extended periods. As such, blocked testing might artificially inflate sensitivity to pitch-timbre covariance, allowing listeners to acclimatize to each condition and perform better than what would otherwise be expected.

To more faithfully model listeners’ natural experience with these patterns of covariance, Experiment 4 interleaved Consistent and Reversed trials throughout the experiment rather than blocking them. Interleaving stimuli increases the uncertainty and acoustic variability from one trial to the next, which slows responses in speeded responding tasks [4,69]. Therefore, performance is predicted to be slower overall in Experiment 4 (interleaved testing) than in Experiment 1 (blocked testing). As in previous experiments, Reversed stimuli are still predicted to be labeled more slowly and less accurately than Consistent stimuli. If perceptual sensitivity to pitch-timbre covariance has indeed been inflated by blocked testing, then the differences between Consistent and Reversed pitch labeling will be smaller in Experiment 4 than they were in Experiment 1 as well.

## Experiment 4

### Procedure

Stimuli were still organized into three 80-trial blocks. Within each 80-trial block, 52 Consistent trials (13 repetitions of each of the four unique stimuli) and 28 Reversed trials (seven repetitions of each of the four unique stimuli) were mixed together. This approximated the 2:1 ratio of Consistent:Reversed trials in Experiment 1, to which the present results are being compared. Like Experiments 1 and 2, no trial-by-trial feedback was provided after each response. Trial orders were fixed such that every listener heard the same trial order in the first test block, a new fixed trial order in the second test block, and a new fixed trial order in the third test block (n.b., fixing trial order across listeners was done in the interest of studying performance on the trial-by-trial level. This was not part of the primary analyses reported here, but the results of these analyses are revealed in the General Discussion). These orders were generated pseudo-randomly, edited only to meet the following three restrictions. First, the same pitch was not allowed to occur on more than three consecutive trials. Second, the same instrument was not allowed to be heard on more than three consecutive trials. Third, the same experimental condition was not allowed to be heard on more than five consecutive trials (this only needed to be enforced for Consistent trials, given that they comprised so much more of experiment). Although trials were no longer blocked by experimental condition, grouping the trials still into three blocks was done to allow participants breaks throughout the experiment.

### Results

#### Response time.

As in other experiments, only correct responses were retained (removing 2148 trials, or 20.34% of the total data) and all response times faster than 200 ms were removed (removing 78 trials, or 0.93% of the remaining data). Distributions of response times were positively skewed, so they were log-transformed to achieve normality. Finally, all responses exceeding three times the standard deviation of each listener’s mean response time of the remaining trials were removed (removing 114 trials, or 0.69% of the remaining data).

Linear mixed-effects modeling was used to predict trial-level response times using the fixed effects of condition (factor-coded, with Consistent serving as the default), Gold-MSI musical training scores (mean-centered), and their interaction. The random effects structure was built iteratively following the same procedures outlined previously. The final random effects structure included random slopes for condition and random intercepts for participants. Model coefficients are listed in Table 4A.

**Table 4:** Mixed-effects modeling results for Experiment 4 (n.b., models shared fixed effects architectures). (A) Results from the linear mixed-effects model analyzing the logarithm of response times are listed at left; (B) results from the generalized linear mixed-effects model analyzing response accuracy are listed at right. Consistent was the default level of the factor Condition, so all fixed effects either depict or are in reference to those trials.

A) Response Time				Experiment 4	B) Accuracy			
$\hat{\beta }$	SE	t	p		$\hat{\beta }$	SE	*Z*	*p*
2.975	0.018	162.530	<0.001	Intercept	3.107	0.170	18.310	<0.001
0.050	0.009	5.708	<0.001	Condition	−2.272	0.222	−10.238	<0.001
−0.002	0.002	−1.070	0.291	Gold-MSI	0.037	0.018	2.107	0.035
−0.002	0.001	−1.918	0.063	Condition x Gold-MSI	0.093	0.024	3.882	<0.001

The first analysis examined condition-level context. Since experimental conditions were not segregated by block (as in previous experiments) but instead were interleaved, here we examine context at the condition level across the entire test session. Relative to Consistent stimuli (estimated marginal mean response time = 943.76 ms, SE = 39.78), response times were significantly slower in response to Reversed stimuli (estimated marginal mean response time = 1059.42 ms, SE = 49.58).

The second analysis examined session-level context. Session-level analyses tested whether and how response times varied as a function of Consistent and Reversed stimuli being blocked (Experiment 1) versus interleaved (Experiment 4). Responses across both experiments were pooled and analyzed in mixed-effect models with fixed main effects of Condition (Consistent set as the default), Gold-MSI musical training score (mean-centered), and Experiment, and all interactions between these terms. Mean response times were significantly slower in Experiment 4 for both Consistent stimuli ($\hat{\beta }$ = 0.10, *t* = 3.44, *p* < .001) and Reversed stimuli ($\hat{\beta }$ = 0.10, *t* = 3.08, *p* < .005). However, the rela*t*ionship between these response times did not significantly change across experiments (Condi*t*ion-by-Experiment interaction: $\hat{\beta }$ = 0.002, *t* = 0.10, *p* = .92).

**Fig 5:**
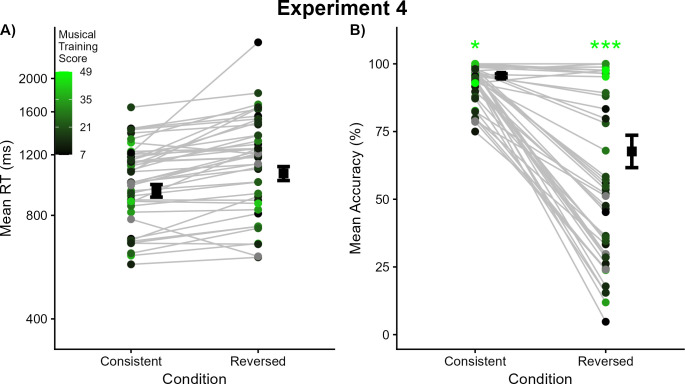
Results from Experiment 4. (A) Each dot represents the mean response time for a given listener in that experimental condition; each listener’s means across conditions are connected by grey lines. The estimated marginal mean response times for each condition are depicted using black squares, with error bars denoting one standard error. Dots are colored according to each listener’s score on the musical training subscale of the Gold-MSI, with brighter (toward green) colors indicating higher scores and darker (toward black) colors indicating lower scores (see inset legend). Grey dots indicate listeners who did not complete the Gold-MSI questions. (B) Each dot represents the mean accuracy for a given listener in that experimental condition; each listeners’ means across conditions are connected by grey lines. The estimated marginal mean accuracy for each condition is depicted using black squares, with error bars denoting one standard error. Dots are again colored according to each listener’s score on the musical training subscale of the Gold-MSI (the same as in panel A). Asterisks denote a statistically significant influence of musical training scores on performance in that block (* *p* < .05, ** *p* < .01, *** *p* < .001).

The third analysis examined long-term context. Three participants did not begin the Gold-MSI questionnaire; another participant began but did not complete the entire survey. The remaining 40 participants completed the Gold-MSI musical training subscale. The mean score (18.59; SD = 9.66) did not differ from that in previous experiments (independent-samples *t*-test against Experiment 1 participants: *t*_75_ = 0.96, *p* = 0.34). Gold-MSI scores for each participant are illustrated in Fig 5 with brighter colors indicating higher scores. These scores were not predictive of response times on Consistent trials ($\hat{\beta }$ = −0.002, *t* = −1.07, *p* = .29) and only trended in that direc*t*ion for Reversed trials ($\hat{\beta }$ = −0.004, *t* = −1.79, *p* = .08).

#### Accuracy.

As above, all response times faster than 200 ms were removed (removing 146 trials, or 1.38% of the remaining data). A generalized linear mixed-effects model was used to predict correct responses on a trial-by-trial basis. Fixed effects in the accuracy model matched those in the response time model detailed above: Condition (factor-coded, with Consistent serving as the default), Gold-MSI musical training score (mean-centered), and their interaction. The random effects structure included random slopes for Condition and random intercepts for Participants. Model coefficients are listed in Table 4B.

The first analysis examined condition-level context. Mean accuracy across the entire test session was high for Consistent stimuli (estimated marginal mean accuracy = 0.96, SE = 0.007) and significantly lower for Reversed stimuli (estimated marginal mean accuracy = 0.68, SE = 0.060).

The second analysis examined session-level context. Session-level effects were analyzed using mixed-effects models with the same architecture detailed above: fixed main effects of Condition (Consistent set as the default), Gold-MSI musical training score (mean-centered), and Experiment, and all interactions between these terms. Accuracy trended toward decreasing on Consistent trials in Experiment 4 ($\hat{\beta }$ = −0.49, *Z* = −1.83, *p* = .07) relative to Experiment 1. Accuracy did not otherwise significantly change across experiments on Reversed trials or the interaction between trial types ($\hat{\beta }$> −0.55, *Z* > −1.15, *p* > .25).

The third analysis examined long-term context. Gold-MSI scores were positively associated with accuracy on Consistent trials ($\hat{\beta }$ = 0.04, *t* = 2.11, *p* < .05) and on Reversed trials ($\hat{\beta }$ = 0.13, *t* = 4.43, *p* < .0001). This relationship was significan*t*ly stronger on Reversed trials than on Consistent trials, as suppor*t*ed by the significant Condition-by-Gold-MSI interaction in Table 4B.

### Discussion

Experiment 4 assessed perceptual sensitivity to the covariance between pitch and timbre when evidence for and against this relationship was interleaved rather than blocked. Performance again replicated the main pattern of Experiments 1–3, with more accurate pitch labeling when pitch and timbre respected their typical covariance rather than violated it. This result was accompanied by a significant difference in response time, with slower responses on Reversed trials. Interestingly, response times in previous experiments generally followed from practice effects, with faster responses when the third block repeated the stimuli from the first block. Only in Experiment 2 were responses to Reversed stimuli significantly slower than responses to Consistent stimuli, and this might have been due to Reversed stimuli being tested first. Thus, superior pitch labeling of Consistent stimuli does not appear to be restricted to certain portions of the experimental session but endure throughout it.

The primary analyses of interest examined how sensitivity to pitch-timbre covariance changed as a function of interleaved (Experiment 4) versus blocked test trials (Experiment 1). Responses were predicted to be significantly slower in Experiment 4 than in Experiment 1, as interleaving trial types increases the acoustic variability and uncertainty from one trial to the next (as in [4,69]). Results confirmed this prediction, as response times to Consistent trials and to Reversed trials were both significantly slower in interleaved trials than in blocked trials. Analyses also assessed whether overall sensitivity to pitch-timbre covariance was inflated through block testing as compared to interleaved testing. This would appear as a significant Condition-by-Experiment interaction, with a smaller difference between conditions in Experiment 4 than in Experiment 1. This interaction was not statistically reliable. However, it must be noted that this is a between-subjects comparison. Future research should consider a more sensitive test of this question by having the same listener group complete both blocked and interleaved testing (possibly with an unrelated intervening task to mitigate practice effects). Thus, interleaving Consistent and Reversed trials slowed pitch labeling, but did not introduce any synergistic effects relative to blocked testing.

Listeners’ musical training played a slightly different role in Experiment 4. In Experiments 1 and 2, Gold-MSI musical training scores were significant predictors of accuracy in every testing block, with stronger associations on Reversed trials than Consistent trials. In Experiment 3, upon the introduction of feedback, the predictive relationship maintained for Reversed trials but became unreliable for Consistent trials. Here, musical training followed the patterns observed in Experiments 1 and 2. Across all experiments, the positive relationship of musical training and pitch labeling does not appear to be entirely global but is more nuanced depending on the experimental factors at play, such as the presence of feedback and the type of pitch-timbre associations being tested.

While not directly designed to measure it, the increased difficulty from interleaving stimuli draws on the twin pillars of informational masking: stimulus dissimilarity and stimulus uncertainty [70–73]. In the blocked testing format of Experiments 1–3, each block presented stimuli that were all similar in terms of whether they respected (Consistent blocks) or violated (Reversed blocks) typical pairings of pitch and timbre. Further, each instrument played only one note in a given block, meaning listeners were only exposed to four unique stimuli at a time (in Consistent blocks, both bright timbres were playing the higher note while both dark timbres were playing the lower note, and vice versa in the Reversed block). This arrangement restricted stimulus uncertainty. In the interleaved testing format of Experiment 4, stimuli were more dissimilar to each other than before (Consistent and Reversed trial types mixed together in quasi-random order) and stimuli were far more uncertain (each instrument played two notes, one of which obeyed patterns of covariance and one which violated it, with no way of knowing which condition and instrument would be tested in next). While none of the pitches nor instruments heard were novel in Experiment 4, these informational factors contributed to slower response times as compared to Experiment 1.

## General discussion

The sensory environment is filled with statistical structure. According to the efficient coding hypothesis [18–19], sensation and perception become attuned to this structure to maximize processing efficiency. One instance of perception seizing upon structure in the acoustic environment is the covariance between musical pitch and timbre: darker timbres (less high-frequency energy) are typically associated with lower pitches and brighter timbres (comparatively more high-frequency energy) are associated with higher pitches [12,26–30,32,37]. This relationship was the focus of four pitch-labeling experiments which sought to characterize and parameterize this perceived covariance more fully. In every experiment, pitch labeling was predicted to be superior when pitch and timbre obeyed their habitual patterns of covariance (Consistent condition) as opposed to when they were violated (Reversed condition). Here we assess the contributions of different timescales of context, the particular stimuli chosen, the role of the task, the nature of musical background, and the statistical structure being tested.

### Timescales of context

The present experiments explored how various timescales of perceptual context shape sensitivity to this pitch-timbre covariance: block level (differences in performance across blocks), session level (performance as a function of testing block order or trial sequencing, contrasted across experiments), and longer-term experience (musical training background). Given the multiplicity of timescales that influence perception most generally, the base prediction stated above was extended to predict superior processing of Consistent sounds over Reversed sounds on each of these timescales.

#### Block level.

Pitch labeling in Reversed blocks was repeatedly less accurate than that in Consistent blocks, whether Reversed stimuli were tested in the second block (Experiment 1), tested in both the first and third blocks (Experiment 2), tested throughout the experiment alongside Consistent trials (Experiment 4), or with feedback (Experiment 3). Even the highest performance level observed across four experiments (proportion correct of 0.95 in Experiment 3) was still inferior to performance in the Consistent blocks of that experiment (0.97–0.98). This poorer accuracy was complemented by slower response times when both conditions were interleaved throughout the experiment (Experiment 4) or when Reversed stimuli were tested first (Experiment 2). Otherwise, mean response times tended to follow practice effects, with faster response times in the third testing block (which repeated the stimuli from the first block) than the first block (Experiments 1–3).

#### Session level.

Alterations to experimental designs provided the longer temporal context of the experimental session for understanding listener performance. In all cases, results from Experiment 1 were taken as the reference point, as each subsequent experiment tested a permutation of its design. The first test of session-level context clarified how performance should be viewed. Changing the order of testing blocks in Experiment 2 demonstrated that listeners were tracking stimulus statistics (maintaining superior performance for Consistent stimuli relative to Reversed stimuli) and not merely exhibiting pure practice effects (superior performance in whichever condition was tested first over whichever condition was tested second). The second manipulation of session-level context was predicted to improve performance, especially for Reversed stimuli. Adding feedback resulted in higher accuracy for Reversed stimuli at levels approaching those for Consistent stimuli (Experiment 3). Other changes to the experimental design increased difficulty relative to that in Experiment 1. The inferior performance for Reversed stimuli was exacerbated when they were tested first and the Consistent block tested second in Experiment 2. Interleaving Reversed and Consistent trials throughout the experiment also increased task difficulty, as revealed by significantly slower response times for both conditions relative to those reported in Experiment 1.

#### Long-term level.

Given that musicians’ performance in pitch perception tasks is widely documented to exceed that of nonmusicians [e.g., 65–67], the long-term context of musical training was expected to facilitate performance in the current pitch labeling tasks. Rather than recruiting listeners on the basis of (extreme amounts of) musical training, training was allowed to vary freely (as it does across the general population). Across four samples, musical training subscores of the Gold-MSI [42] were remarkably consistent, with means ranging from 18.59 to 22.62 out of 49 (see S1 Fig). In every experiment, Gold-MSI scores were significant predictors of accuracy in at least one experimental condition. In Experiments 1, 2, and 4, musical training scores were predictive of accuracy in every block, with stronger associations observed for Reversed stimuli than for Consistent stimuli. Upon the introduction of feedback in Experiment 3, musical training scores were still predictive of pitch labeling accuracy for Reversed stimuli but not for Consistent stimuli. In other words, feedback improved perception of Consistent stimuli to the point where higher musical training scores were no longer reliably associated with better performance. The challenge of perceiving Reversed pairings of pitch and timbre was lessened but still persisted, moreso for listeners with less musical training who are more apt to confuse the two [9,38,39]. As discussed previously, this perceptual context differs from the others in that it is an active experience above and beyond passive exposure to instrument pitches and timbres (which listeners receive with or without musical training). Thus, the long-term context of musical training shares a strong relationship with pitch perception when timbre is violating their common covariance (significant relationship in every Reversed block/ condition across all experiments), and a reliable but comparatively weaker relationship when timbre respects its typical covariance with pitch (significant relationships in most but not all Consistent blocks/ trials).

#### Other pertinent timescales of context for perception.

Block-level, session-level, and long-term timescales were of particular interest because they suited the pitch labeling experimental designs. Other timescales of context inform perception and performance as well, some of which are briefly reviewed here. The shortest possible timescale for context is (near-) immediate, or what is termed “one-shot learning.” In computer vision, prior knowledge from previous learned categories can accelerate accurate classification in a new category from few or even one exemplar [e.g., 74−75]. Similar behavior has been reported in humans, as seeing or hearing a single novel token allows perceivers to recognize key features and generate new tokens based on inferences about the structure of the category [76–79]. These processes are sufficiently robust that 6-to-12-month-old infants outperform artificial neural networks in object classification following habituation to a single novel exemplar [80].

A second timescale of interest is that of other stimuli presented on a given trial. Surrounding sounds play an incredibly important role in auditory perception, especially in perception of speech (for review see [81]). In the present experiments, only one sound was presented on each trial. Other studies have examined pitch-timbre interdependence when presenting multiple sounds on each trial, forming an acoustic context for perception of the target sound. These studies reported a weaker relationship between pitch and timbre than when sounds were presented in isolation [8,13,82]. But, perception of one sound among many may utilize different tasks and/or invoke different memory demands than those involved with perception of isolated sounds. Possible task effects are discussed later in the General Discussion.

A third relevant timescale is that of trial-to-trial relationships within a testing block. Mean performance in a block offers an end-state perspective on behavior, but not in how performance arrived at that particular mean (e.g., gradually, sharply, ballistically, randomly). While not part of the primary analyses, exploratory analyses were also conducted on the present results to examine the shorter timescale of trial-to-trial behavior. These were captured using the fixed effect of trial, which estimates the linear regression slope for that dependent measure across trials, and how this slope varied across blocks (trial-by-block interactions). Clear overarching patterns were observed, supplementing the results using block-level means reported above (which are annotated here in parentheses). For response times, trial-by-trial responses became significantly faster throughout every block, but the rate of this decrease became shallower in the final block of each experiment (when mean response time was significantly faster with repeated stimuli) relative to the first block (S2 Fig). When block structure was removed in Experiment 4 and conditions were interleaved, the response time slope was steeper (and mean response time higher) for Reversed stimuli than for Consistent stimuli. For accuracy, responses became significantly more accurate throughout Reversed blocks (where mean accuracy was comparatively lower) but did not exhibit significant improvement throughout Consistent blocks (where mean performance was near/at ceiling levels; S3 Fig). Finally, for both response times and accuracy, shallower slopes in Experiment 4 (and elevated mean response times) indicated increased difficulty when stimuli were interleaved relative to the blocked testing order of Experiment 1. All analyses are documented in the Open Science Framework repository for this research: https://osf.io/fpj8q/.

The final relevant timescale of context is the longest one available to the listener, their lifetime of perceptual experience. Pitch and timbre are essential sound qualities not only in perception of music but for perception of all sounds. One’s lifetime of experience with pitch, timbre, and the degree to which they covary is highly germane to any task where sensitivity to their relationship is assessed. Finally, perception of musical sounds is clearly not the only situation in which perceivers rely on various concurrent timescales of context. For a review of these tenets in language processing, see [83], who extend these timescales beyond one’s lifetime of perceptual experience to include the historical context of language evolution as well.

### Efficient coding and auditory perception

The present results mark an advance in applications of the efficient coding hypothesis to auditory perception. Reports of efficient coding in auditory neural responses are plentiful [84–88], but demonstrations of efficient coding in auditory perception are far fewer. Of those published, these studies tend to follow two main threads. In the first thread, studies identify certain statistical regularities in the acoustic environment then confirm perceptual sensitivity to them [24,89–91]. In the second thread, a statistical regularity is created *de novo* and perception rapidly learns it [20–22]. However, in both of these threads, the listeners and their perceptual experience with the stimulus regularity are treated relatively uniformly. The possibility that a statistical regularity in the auditory environment might have differential effects on perception has not yet been explored. Here, listeners varying in their musical training exhibited differential responses to patterns of covariance between musical pitch and timbre. Listeners reporting less musical training tended to perform very differently when stimulus statistics were altered, with superior performance on Consistent trials than on Reversed trials. Conversely, listeners reporting more musical training tended to perform more similarly across both conditions. These patterns of performance held whether trial-by-trial feedback was provided (as in Experiment 3) or not (Experiments 1, 2, and 4), speaking to the differences in participants’ long-term perceptual experience (vis a vis musical training) and not solely short-term learning effects. For these listeners, this result might be interpreted as sensitivity to *their* experienced distributions of statistical inputs, where pitch and timbre are heard in these reversed pairings relatively more often during musical listening and/or training. This would cohere with extensive literature on superior pitch perception by listeners with more musical training (the hallmark finding of the “musician advantage” [e.g., 65–67]). This highlights the need to incorporate listener factors into investigations of efficient coding in auditory perception, as experience with a given statistical regularity cannot be assumed to be constant across a sample or a population (also see the section titled Musical Background for more discussion).

### The stimuli

In investigations of pitch-timbre interference and/or covariance, the pitches and pitch intervals tested have varied widely. Here, the pitch labeling task tested utilized a seven-semitone interval (C4 - G4). This interval was selected based on difficulties for some participants (particularly those with less musical training) to accurately label the pitches of tones that spanned only four semitones [11]. Several studies have examined the relationship between pitch and timbre by testing intervals of six or more semitones (F^#^4 - C5 [8]; D4 - G^#^4 [9]; pitches ranging from F3 - E4 [28]). Other studies tested smaller intervals including two or four semitones (among F4, G4, and A4 [38]) or pitch deviations no larger than one-half semitone (50 cent deviation in pitch relative to A3 [30]). A recent study by McPherson and McDermott [27] reported perceptual benefits of pitch-timbre covariance across a series of pitch intervals spanning 0.11–9 semitones. Studies employing nonmusical sounds were similarly variable in the pitches and pitch changes tested, comparing 900 and 950 Hz [7], comparing two frequencies selected from 300–600 Hz that shared a 1.04:1 ratio [13], comparing 200 Hz to a frequency ranging from 136–264 Hz [29], comparing 200 Hz to various frequencies determined by each listener’s discrimination threshold [26], and detecting changes among 318, 337, 357, and 378 Hz [17].

Stimulus variability across studies is also evident in the timbres presented. Here, specific recordings of trumpet, oboe, trombone, and tuba were selected to span a wide range of spectral brightnesses (Fig 1). This range is comparable to those tested previously, which includes tones produced by the trumpet and piano [8–9], the ukulele, pipe organ, cello, oboe, and baritone saxophone [27], the viola and the trumpet [30], and chords played on the guitar, piano, and harpsichord [15]. Other studies have used synthetic timbres to increase experimental control over stimuli spectra. These synthetic timbres spanned an even wider range of brightnesses, with stimuli including pure tones [13], harmonic complexes with a restricted range of harmonics (≤6 harmonics; [13,29,39]) or a wider range of harmonics (>8 harmonics; [7,10,28]), and wideband harmonic complexes with one prominent spectral peak [26,39]. On the whole, pitch and timbre display perceptual interdependence across many different pitches, pitch intervals, and timbres. At the same time, this relationship is not a universal one, as it may depend on the instrument in question [36–37].

Instruments were assigned to ‘dark’ and ‘bright’ levels of spectral brightness relative to the other timbres presented before pairing them with congruent or incongruent pitches (Fig 1). The tuba and trumpet marked relative extremes of brightness, but the intermediate brightnesses of the trombone and oboe require closer consideration. While the trombone spectrum is certainly darker than the spectra we labeled ‘bright’ (trumpet and oboe), at the same time its spectral profile is brighter than that of the other instrument we labeled ‘dark’ (tuba). Similarly, while the oboe spectrum is certainly brighter than the spectra we labeled ‘dark’ (trombone and tuba), it is darker than that of the other instrument we labeled ‘bright’ (trumpet). We did not have a priori predictions about instrument-specific effects in these pitch labeling tasks, but one cannot rule out differences in responses to extreme and less-extreme timbres at a given level of brightness. To examine potential instrument-specific effects, supplementary analyses were conducted using the final versions of mixed-effects models reported above with an added fixed effect of instrument and their interactions with block. All of the following analyses are documented in the Open Science Framework repository for this research: https://osf.io/fpj8q/.

To analyze perception of darker timbres, tuba was set as the default level of instrument to assess whether responses differed from those to the trombone. Analyses focused on differences across the first two blocks (one Consistent block and one Reversed block apiece in Experiments 1–3) or throughout the experiment (Experiment 4). For Consistent trials (all experiments), trombones were repeatedly labeled slower and less accurately than tubas (S4 S5 Figs). This conveys a degree of perceptual confusion when hearing the less-dark trombone timbre relative to the extreme-dark tuba timbre. However, for Reversed trials (Experiments 1, 2, and 4), trombones were labeled more accurately than tubas. In these cases, the comparatively brighter timbres of trombones did not suffer as much from pitch-timbre confusions as tubas did. These differences were supported by significant interactions between these instruments and block (or in the case of Experiment 4, instrument-by-condition) in every experiment for both response time data and accuracy data.

To analyze perception of brighter timbres, trumpet was set as the default level of instrument to assess whether responses differed from those to the oboe. Instrument effects were present, but not as pronounced as they were between the tuba and trombone (S4 S5 Figs). In every experiment, responses to the oboe were more accurate than to the trumpet on Reversed trials. Also, the difference in accuracies for trumpet and oboe tended to be larger on Reversed trials than on Consistent trials (Experiments 1, 2, 4). These both echo the finding above that less-extreme timbres were less susceptible to pitch-timbre confusions than more extreme timbres. For response times, listeners responded to oboes more quickly than trumpets on Reversed trials of Experiment 4 (and trended in the opposite direction for Consistent trials, with slower responses to oboes), which again conveys a degree of resilience to pitch-timbre confusions for less-extreme timbres. Finally, significant interactions between these instruments and block (or again in the case of Experiment 4, instruments by condition) were observed for accuracy data of Experiments 1, 2, and 4, and these interactions were significant for response time data only in Experiment 4. Overall, these instrument-specific effects do not weaken the results or conclusions from the present experiments, but instead highlight the continuous nature of spectral brightness and raise valuable considerations for stimulus selection in future studies.

### The task

The choice of behavioral task plays an important role in the relationship between musical pitch and timbre. Demonstrations of perceptual interference between pitch and timbre and/or the covariance between them have employed tasks including labeling (the present experiments; [7–8]), discrimination [26–27], rating the size of a pitch interval [28], rating pitches as in-tune or out-of-tune [10], pleasantness ratings [32], associating pitch height with spatial height [35], or various forms of change detection across successive sounds [9,15,29,38]. Studies reporting less perceptual interdependence between pitch and timbre had listeners detect changes in the midst of sequences of other sounds [8] or across an intervening set of sounds [13,82]. Thus, the perceptual interdependency between pitch and timbre does not solely follow the number of tones presented on a given trial (as originally suggested by [10]), but interacts with what listeners are being asked to do.

The relationship between the present pitch labeling and Garner interference paradigms [92–93] also merits closer examination. Garner interference paradigms have a long and rich history of examining perceptual dependency between stimulus dimensions (for review see [94]), including the dependency between pitch and timbre [7–8]. These dependencies are often measured as increases in response times (and/or decreases in accuracy) when irrelevant stimulus variation challenges labeling the stimulus property of interest (e.g., when timbre is varying during a pitch labeling task) relative to when no extraneous variability is present (i.e., the baseline). Typically, performance is facilitated when the irrelevant stimulus dimension is covarying with the dimension of interest (‘redundancy gain’) rather than varying randomly. Melara and Marks [7] proposed that the congruency of acoustic variability across dimensions (specifically, the direction of this correlation) could be specifically encoded at a later stage in processing. This bears directly on the present studies, as performance was facilitated when pitch and timbre dimensions covaried in accordance with their natural pairings but was challenged when they covaried in the competing direction. Curiously, Krumhansl and Iverson [8] did not observe performance gains related to the direction of the correlation, reporting mean response times of 304 ms when sounds followed these patterns of covariance (darker piano timbre with 370 Hz, brighter trumpet with 523 Hz) and 307 ms when sounds violated them (piano with 523 Hz, trumpet with 370 Hz). One possible reason for this discrepancy was that Krumhansl and Iverson [8] exclusively tested musicians (minimum 5 years of training ending not more than 3 years before participating in the study). As the present findings attest, musical training provides a degree of resilience to Reversed stimuli, with some of the more musical participants performing equally well in both blocks (see bright green dots in Figs 2b, 3b, 4b). Nevertheless, as reviewed above, pitch labeling and Garner interference studies are two of many methodological options that can illuminate perceptual sensitivity to the covariance between pitch and timbre.

### Musical background

Scores of studies have examined the so-called “musician advantage”, testing instances of superior perceptual faculties for individuals with musical training compared to individuals without musical training. Primary among these findings is superior pitch perception for musicians (e.g., [65–67]). However, musical training is a continuous variable; treating it as a binary variable can introduce serious statistical challenges [41]. Here, musical training was measured continuously using the musical training subscale of the Gold-MSI [42]. In every experiment, significant relationships were observed between musical training scores and mean accuracy in pitch labeling. Increased exposure to various pitch and timbre combinations, including less typical ones, could underlie such an effect. For example, formal musical training at the university level increases listening exposure both implicitly (as in listening in practice, rehearsal, or performance settings) and explicitly (as in orchestration or aural skills classes). Together, this reliable statistical relationship between training and accuracy reaffirms the influence of individual considerations on pitch tasks beyond group level influences.

Listeners’ musical training served as a long-term context that influenced their performance on the pitch labeling tasks tested here. However, musical training is not the only means by which listeners could excel in such a task. Individuals termed “musical sleepers” [95] exhibited similarly superior musical perceptual performance as musicians but without the history of extensive musical training. There exist myriad reasons why listeners without musical training might perform well on such tasks. One recent report revealed that audio engineers, who have extensive auditory perception experience, performed similarly to experienced musicians and superior to control participants (who did not have musical training nor audio engineering experience) across a variety of perceptual tasks [96]. Future research in this area would benefit from taking a broader approach to assessing musical (and perceptual) background, such as testing the full Gold-MSI questionnaire to measure overall musical sophistication rather than subscales that target musical training specifically.

### The statistics being learned

Disagreement exists as to which statistical properties in the environment are most formative for perception. Probability of presentation has been argued to be fundamental to speech perception [97–100], sometimes arguing that other statistical regularities play at best secondary roles in development [101]. Other investigations of the perception of nonspeech sounds (including the present experiments using music sounds) offer evidence where covariance among sound features influences perception more than probability density. In Experiment 2, Reversed trials were tested twice as often as they were in Experiment 1, yet the accuracy of pitch labeling did not improve. In other words, the (violation of) covariance between pitch and timbre influenced perception more strongly than the increased number of test trials. This result echoes Stilp and Kluender [21], where listeners discriminated novel sounds whose acoustic properties covaried strongly with one another (ratio of attack/decay covarying with spectral brightness). Sound discriminability was dictated by acoustic property covariance, even when the lone sound pair that violated this pattern was tested ten times more often than any sound pair supporting it. In subsequent experiments, as a single sound pair increasingly violated the overarching pattern of covariance, its discriminability improved and ultimately far surpassed that of sounds which respected the covariance [22]. While it has not been directly tested, it is unlikely that the relative perceptual salience of statistical relationships in the acoustic environment changes depending on the domain (e.g., prevalence of probability density and covariance among features in speech vs. music). A unified account of how listeners encode different statistical regularities concurrently will be invaluable for understanding perceptual development and encoding in general.

Probability of presentation and covariance among features are but two of many statistical regularities in the sensory environment. Exploration of additional regularities (including but not limited to transitional probabilities and nonadjacent dependencies) have long been a focus in the literature on statistical learning [102–105]. A primary focus of statistical learning examines how learners exploit statistical regularities (often tested in the visual and/or auditory domains) to acquire and develop larger systems such as language. However, the goals of studying statistical learning are not that dissimilar to those of efficient coding – in both cases, understanding the sensitivity to reliable associations in the sensory environment is of fundamental importance, as it underlies efficient processing as well as higher-level perceptual and cognitive organization. While the destinations may differ in their complexity, the routes servicing them share considerable overlap.

## Supporting information

S1 Text Distributions of Gold-MSI musical training scores in each experiment. Each panel depicts the distribution of Gold-MSI Musical Training subscale scores in that given experiment. Bin size is set to 1 so that all scores are easily visible, ranging from a minimum score of 7 and a maximum score of 49.
(PDF)

S2 Text Effects of trial number on response times. Dots indicate the grand mean of listeners’ mean response times for each trial in each block (panels), organized by experiment (columns). Red dots depict mean response times to Consistent trials, and grey dots depict mean response times to Reversed trials. Error bars denote one standard error of the mean, calculated across each listeners’ mean response time on that given trial. As in other response time analyses, only trials where the correct response was provided are included. Linear mixed-effects regressions were used to predict response times on each trial in each block (with each block set as the default level of the fixed effect of block in turn). Since Experiment 4 was not divided into blocks (thereby separating Consistent trials from Reversed trials, as was done in previous experiments), regression models analyzed performance as a function of overall trial number in the experiment (‘Trialoverall’ on the x-axis; out of 240). In these models, the intercept and the regression coefficient for the fixed effect of trial number were used to calculate the slope in terms of how much faster responses were estimated to get on each successive trial. These slopes are labeled at the bottom of each panel. Every slope significantly differed from zero, such that response times decreased throughout each block of each experiment. Experiments 1 and 2 patterned similarly: slopes in the first two blocks did not differ from each other, but both were significantly steeper (i.e., response times decreased at a faster rate) than the slope in the final block. In Experiment 3, all slopes were significantly different from each other, and response times decreased at shallower rates in each successive block. In Experiment 4, both slopes significantly differed from zero but were significantly steeper for Reversed trials.
(PDF)

S3 Text Effects of trial number on accuracy Dots indicate the grand mean of listeners’ mean accuracy for each trial in each block (panels), organized by experiment (columns). Red dots depict mean accuracy for Consistent trials, and grey dots depict mean accuracy to Reversed trials. Error bars denote one standard error of the mean, calculated across each listeners’ mean response time on that given trial. Generalized linear mixed-effects regressions were used to predict accuracy on each trial in each block (with each block set as the default level of the fixed effect of block in turn). Since Experiment 4 was not divided into blocks (thereby separating Consistent trials from Reversed trials, as was done in previous experiments), regression models analyzed performance as a function of overall trial number in the experiment (‘Trialoverall’ on the x-axis; out of 240). In these models, the intercept and the regression coefficient for the fixed effect of trial number were used to calculate the slope in terms of how much more accurate responses were estimated to get on each successive trial. These slopes are labeled at the bottom of each panel. It bears note that mixed-effect models overestimate mean accuracy on Reversed trials (higher estimated intercept than the data seem to warrant), but seem to preserve the slope (i.e., rate of improvement throughout the block). In Experiment 1, accuracy slopes did not differ from zero in the first and third (Consistent) blocks, consistent with ceiling performance. The slope in the second block (Reversed) was significant and positive, indicating increasing accuracy throughout the block. This slope was significantly greater than slopes in the first and third blocks. In Experiment 2, the accuracy slope trended toward being positive in the first (Reversed) block, which would follow from the improvement observed throughout the Reversed block in Experiment 1. However, slopes were unexpectedly significantly negative in the second block (Consistent) and did not differ from zero in the final (Reversed) block. The slope in the second block significantly differed from the other blocks, which did not differ from each other. Slopes in Experiment 3 followed the overall pattern observed in Experiment 1 (not differing from zero in the first and final [Consistent] blocks, significant and positive in the Reversed block). The primary difference from Experiment 1 results was that the significant positive slope for the Reversed block only trended toward significantly differing from the flat slopes in the other blocks. This is likely due to the elevated performance stemming from receiving feedback on each trial. Finally, in Experiment 4, the slope for Consistent trials did not differ from zero, but was significant and positive for Reversed trials.
(PDF)

S4 Text Effects of instruments on response times. Bars depict mean response times for each instrument (± standard error) in the first two blocks of Experiments 1–3 and throughout Experiment 4. Response times are included in averaging only for trials with correct responses. Bars are colored according to their organization into ‘dark’ (tuba = black, trombone = grey) and ‘bright’ timbres (trumpet = cyan, oboe = dark cyan). Arrows indicate significant changes in response time across two stimuli in the same level of timbral brightness. Increases in response time are observed in Consistent conditions when moving from the tuba (the extreme level of brightness, paired congruously with pitch in that block) to the trombone (the less-extreme level of brightness). These results are coherent with an increase in pitch-timbre confusions. Generally, decreases in response time in Reversed conditions when moving from the extreme level of brightness (which is paired incongruously with pitch in that block) to a less extreme level would be coherent with a decrease in pitch-timbre confusions. This was observed only in Experiment 4 when moving from the trumpet (the brightest timbre) to the oboe (less bright than the trumpet).
(PDF)

S5 Text Effects of instruments on accuracy. Bars depict mean accuracy for each instrument (± standard error) in the first two blocks of Experiments 1–3 and throughout Experiment 4. Bars are colored according to their organization into ‘dark’ (tuba = black, trombone = grey) and ‘bright’ timbres (trumpet = cyan, oboe = dark cyan). Arrows indicate significant changes in accuracy across two stimuli in the same level of timbral brightness. Generally, decreases in accuracy are observed in Consistent conditions when moving from the extreme level of brightness (which is paired congruously with pitch in that block) to a less-extreme level (from tuba [darkest] to trombone [less dark than tuba]; from trumpet [brightest] to oboe [less bright than trumpet]). These results are coherent with an increase in pitch-timbre confusions. Generally, increases in accuracy are observed in Reversed conditions when moving from the extreme level of brightness (which is paired incongruously with pitch in that block) to a less extreme level, coherent with a decrease in pitch-timbre confusions.
(PDF)
