# Supplementary figures and images for: Multiple timescales of context influence perceptual sensitivity to common pairings of musical pitch and timbre
Source: PLoS One. 2025 Jul 18;20(7):e0328490. doi: 10.1371/journal.pone.0328490 (PMC12273972; doi:10.1371/journal.pone.0328490)

**Experiment 1**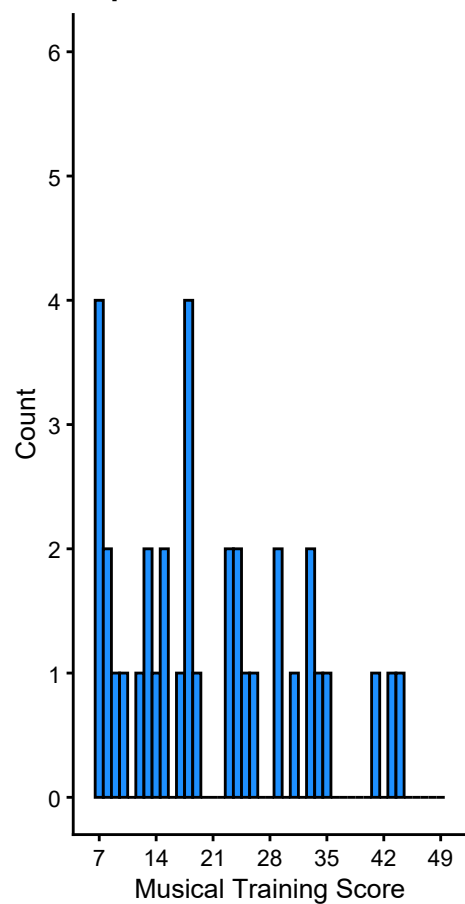**Experiment 2**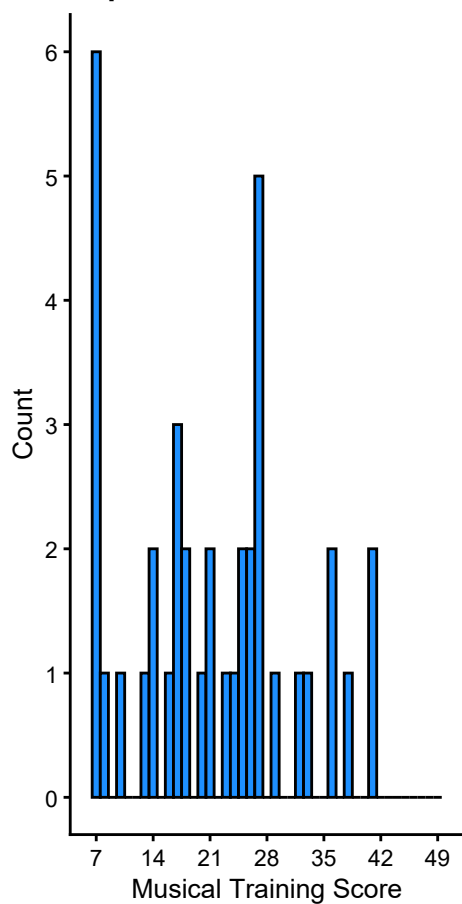**Experiment 3**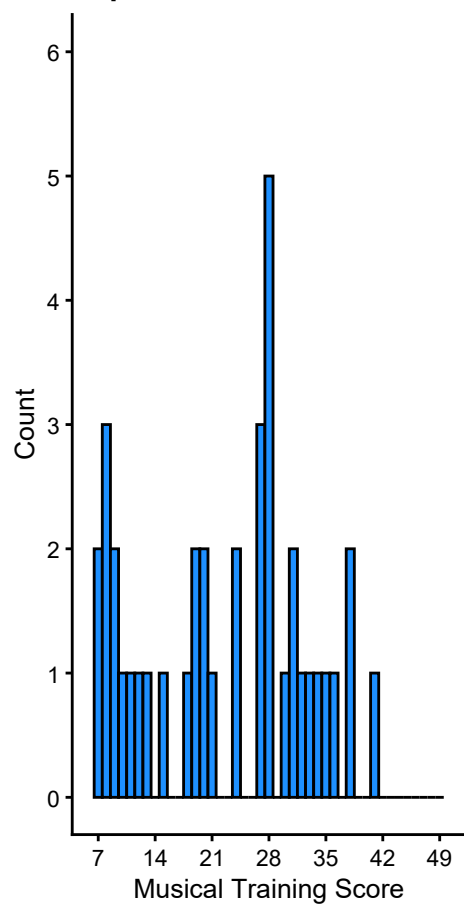**Experiment 4**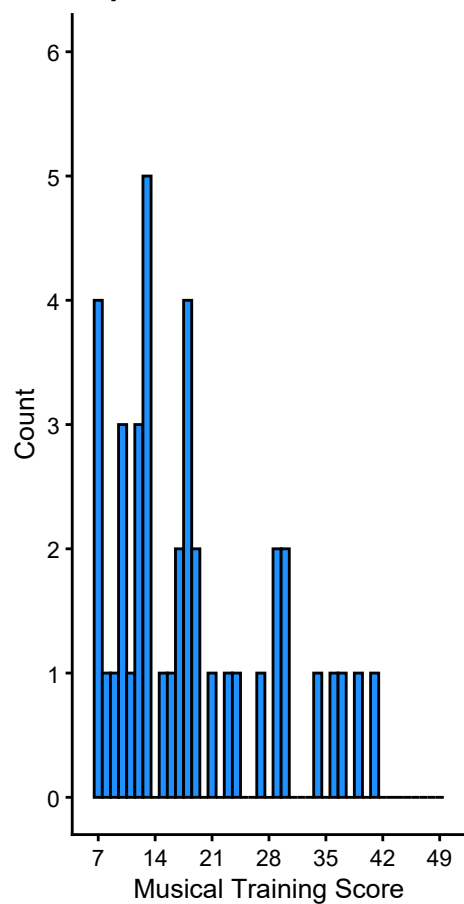

Supplement: S1 Text — Each panel depicts the distribution of Gold-MSI Musical Training subscale scores in that given experiment. Bin size is set to 1 so that all scores are easily visible, ranging from a minimum score of 7 and a maximum score of 49. (PDF) [file pone.0328490.s001.pdf]

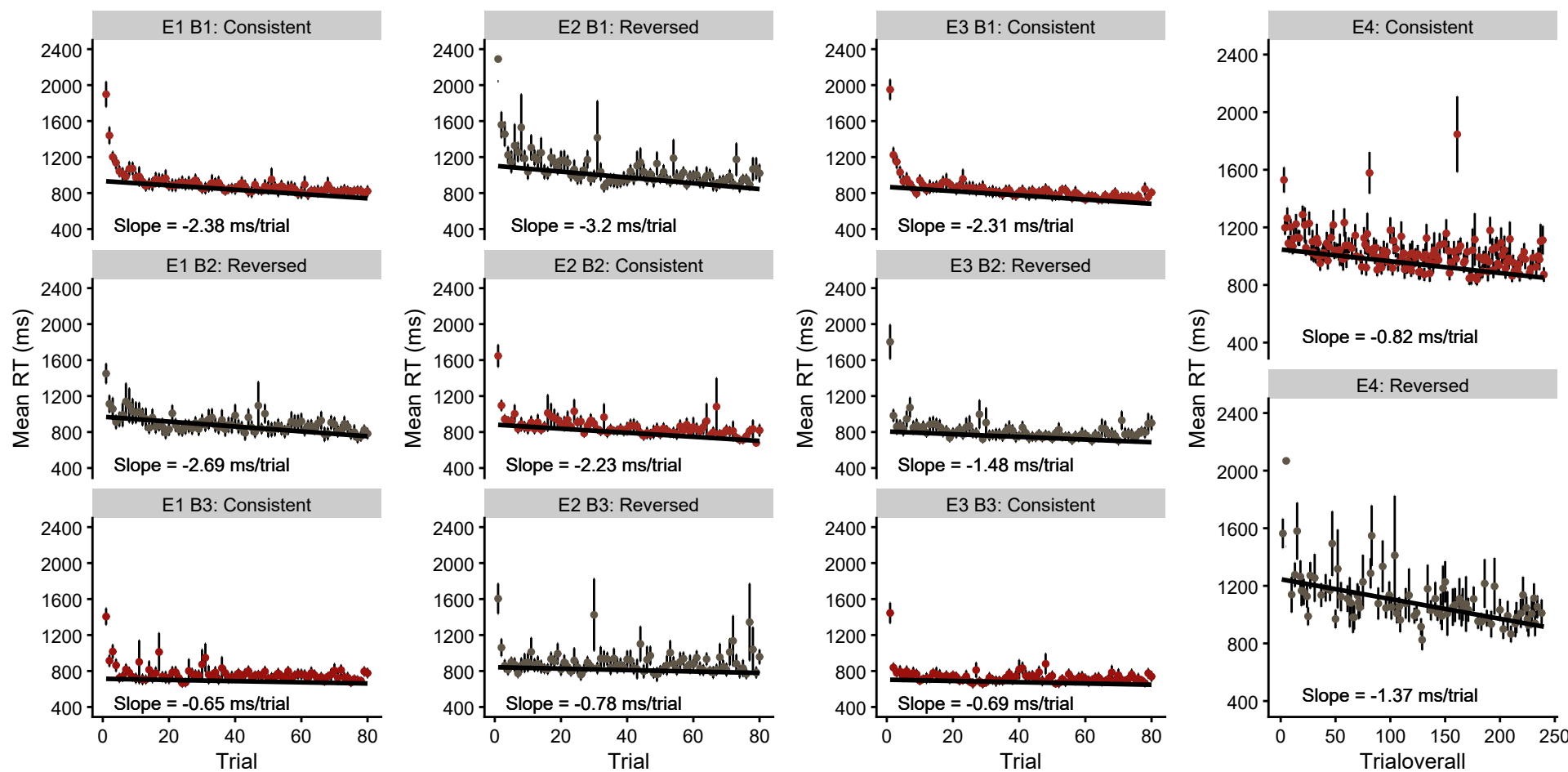

Supplement: S2 Text — Dots indicate the grand mean of listeners’ mean response times for each trial in each block (panels), organized by experiment (columns). Red dots depict mean response times to Consistent trials, and grey dots depict mean response times to Reversed trials. Error bars denote one standard error of the mean, calculated across each listeners’ mean response time on that given trial. As in other response time analyses, only trials where the correct response was provided are included. Linear mixed-effects regressions were used to predict response times on each trial in each block (with each block set as the default level of the fixed effect of block in turn). Since Experiment 4 was not divided into blocks (thereby separating Consistent trials from Reversed trials, as was done in previous experiments), regression models analyzed performance as a function of overall trial number in the experiment (‘Trialoverall’ on the x-axis; out of 240). In these models, the intercept and the regression coefficient for the fixed effect of trial number were used to calculate the slope in terms of how much faster responses were estimated to get on each successive trial. These slopes are labeled at the bottom of each panel. Every slope significantly differed from zero, such that response times decreased throughout each block of each experiment. Experiments 1 and 2 patterned similarly: slopes in the first two blocks did not differ from each other, but both were significantly steeper (i.e., response times decreased at a faster rate) than the slope in the final block. In Experiment 3, all slopes were significantly different from each other, and response times decreased at shallower rates in each successive block. In Experiment 4, both slopes significantly differed from zero but were significantly steeper for Reversed trials. (PDF) [file pone.0328490.s002.pdf]

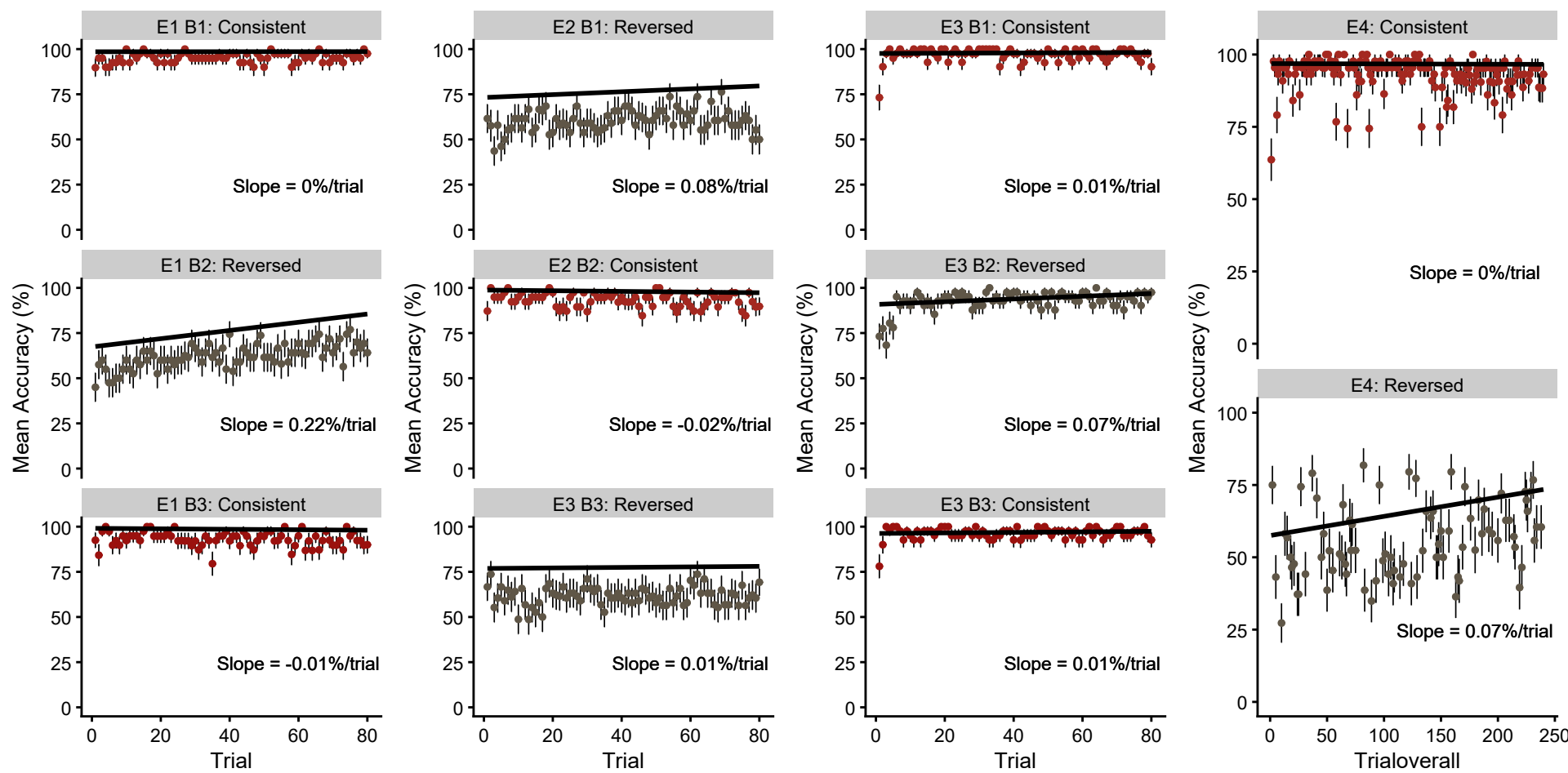

Supplement: S3 Text — Dots indicate the grand mean of listeners’ mean accuracy for each trial in each block (panels), organized by experiment (columns). Red dots depict mean accuracy for Consistent trials, and grey dots depict mean accuracy to Reversed trials. Error bars denote one standard error of the mean, calculated across each listeners’ mean response time on that given trial. Generalized linear mixed-effects regressions were used to predict accuracy on each trial in each block (with each block set as the default level of the fixed effect of block in turn). Since Experiment 4 was not divided into blocks (thereby separating Consistent trials from Reversed trials, as was done in previous experiments), regression models analyzed performance as a function of overall trial number in the experiment (‘Trialoverall’ on the x-axis; out of 240). In these models, the intercept and the regression coefficient for the fixed effect of trial number were used to calculate the slope in terms of how much more accurate responses were estimated to get on each successive trial. These slopes are labeled at the bottom of each panel. It bears note that mixed-effect models overestimate mean accuracy on Reversed trials (higher estimated intercept than the data seem to warrant), but seem to preserve the slope (i.e., rate of improvement throughout the block). In Experiment 1, accuracy slopes did not differ from zero in the first and third (Consistent) blocks, consistent with ceiling performance. The slope in the second block (Reversed) was significant and positive, indicating increasing accuracy throughout the block. This slope was significantly greater than slopes in the first and third blocks. In Experiment 2, the accuracy slope trended toward being positive in the first (Reversed) block, which would follow from the improvement observed throughout the Reversed block in Experiment 1. However, slopes were unexpectedly significantly negative in the second block (Consistent) and did not differ from zero in the fi [file pone.0328490.s003.pdf]

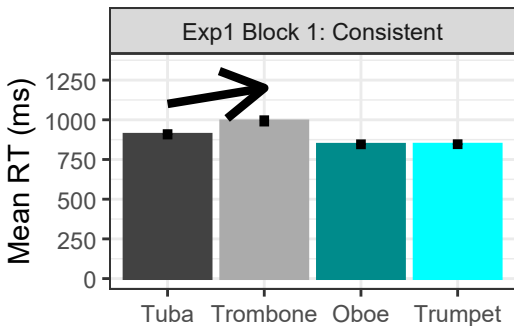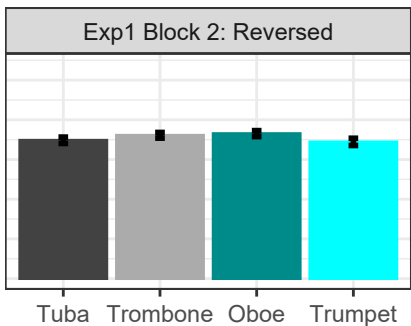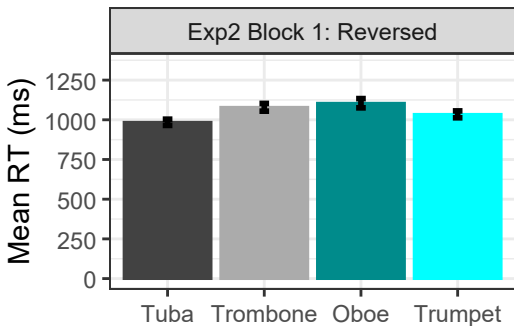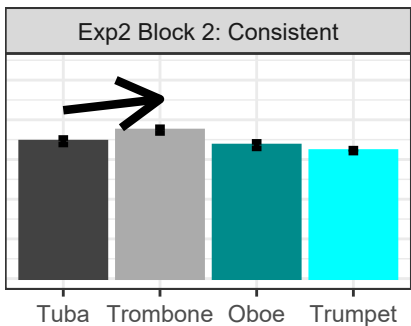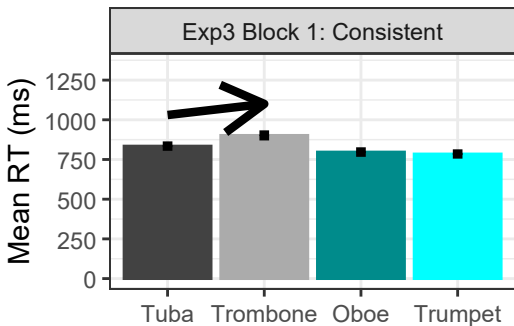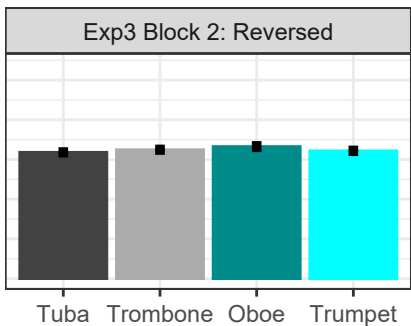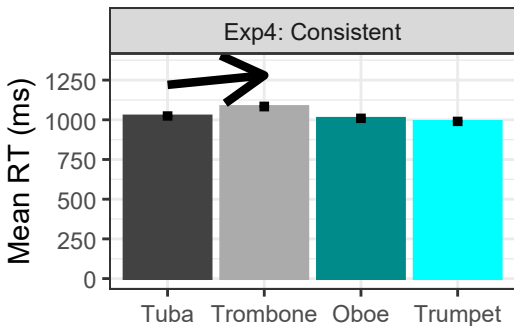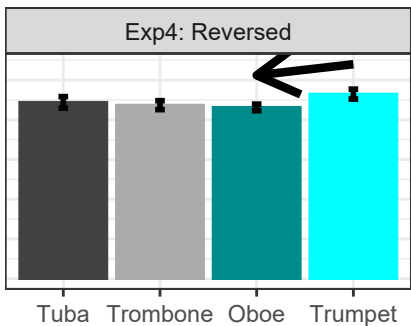

Instrument

Supplement: S4 Text — Bars depict mean response times for each instrument (± standard error) in the first two blocks of Experiments 1–3 and throughout Experiment 4. Response times are included in averaging only for trials with correct responses. Bars are colored according to their organization into ‘dark’ (tuba = black, trombone = grey) and ‘bright’ timbres (trumpet = cyan, oboe = dark cyan). Arrows indicate significant changes in response time across two stimuli in the same level of timbral brightness. Increases in response time are observed in Consistent conditions when moving from the tuba (the extreme level of brightness, paired congruously with pitch in that block) to the trombone (the less-extreme level of brightness). These results are coherent with an increase in pitch-timbre confusions. Generally, decreases in response time in Reversed conditions when moving from the extreme level of brightness (which is paired incongruously with pitch in that block) to a less extreme level would be coherent with a decrease in pitch-timbre confusions. This was observed only in Experiment 4 when moving from the trumpet (the brightest timbre) to the oboe (less bright than the trumpet). (PDF) [file pone.0328490.s004.pdf]

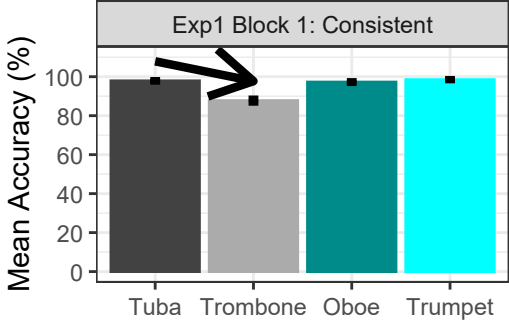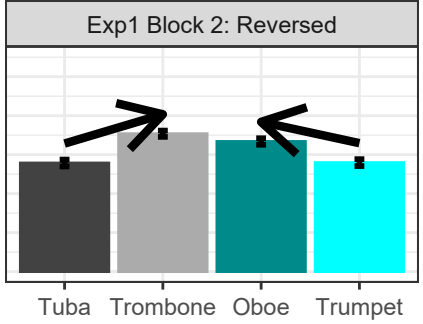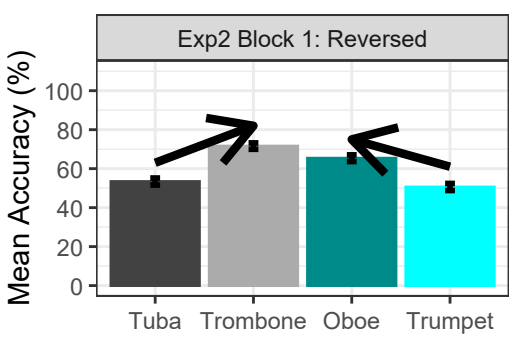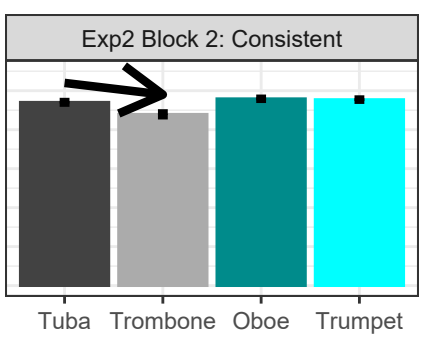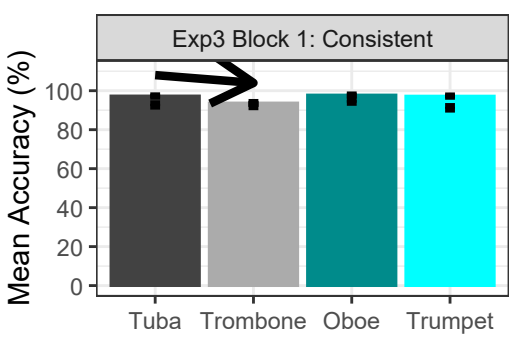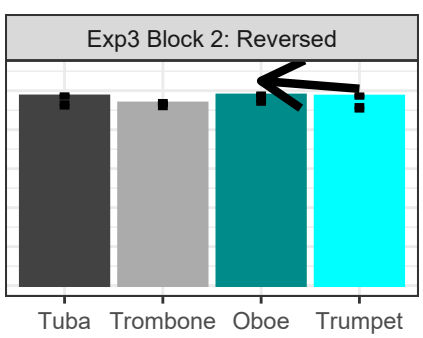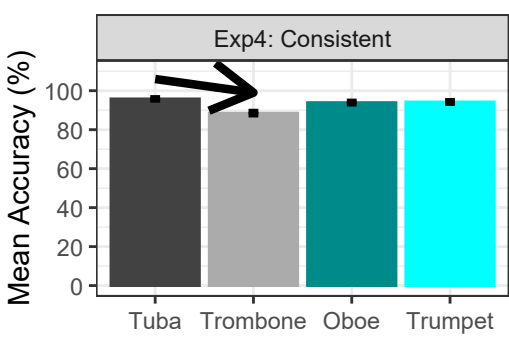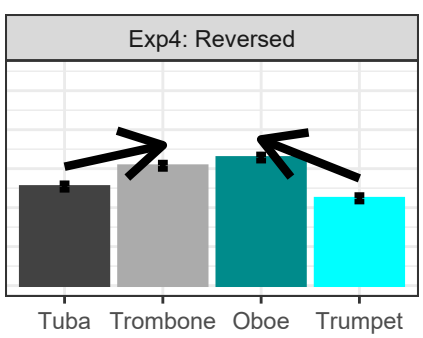

Instrument

Supplement: S5 Text — Bars depict mean accuracy for each instrument (± standard error) in the first two blocks of Experiments 1–3 and throughout Experiment 4. Bars are colored according to their organization into ‘dark’ (tuba = black, trombone = grey) and ‘bright’ timbres (trumpet = cyan, oboe = dark cyan). Arrows indicate significant changes in accuracy across two stimuli in the same level of timbral brightness. Generally, decreases in accuracy are observed in Consistent conditions when moving from the extreme level of brightness (which is paired congruously with pitch in that block) to a less-extreme level (from tuba [darkest] to trombone [less dark than tuba]; from trumpet [brightest] to oboe [less bright than trumpet]). These results are coherent with an increase in pitch-timbre confusions. Generally, increases in accuracy are observed in Reversed conditions when moving from the extreme level of brightness (which is paired incongruously with pitch in that block) to a less extreme level, coherent with a decrease in pitch-timbre confusions. (PDF) [file pone.0328490.s005.pdf]
